# Synthesis of Cobalt Hydroxychloride and Its Application as a Catalyst in the Condensation of Perimidines

**DOI:** 10.3390/molecules31010182

**Published:** 2026-01-04

**Authors:** Cássio Siqueira, Gabriela R. Borges, Fernanda S. Portela, Maria E. Miks, Felipe F. Marques, Gleison A. Casagrande, Sumbal Saba, Rafael Marangoni, Jamal Rafique, Giancarlo V. Botteselle

**Affiliations:** 1 Laboratory of Organic Synthesis and Catalysis (LabSOC), Midwestern Parana State University-UNICENTRO, Guarapuava 85040-167, PR, Brazil; 2 Instituto de Química (INQUI), Universidade Federal do Mato Grosso do Sul-UFMS, Campo Grande 79074-460, MS, Brazil; 3 Laboratory of Sustainable Synthesis and Organochalcogen (LabSO), Instituto de Química (IQ), Universidade Federal de Goiás–UFG, Goiânia 74690-900, GO, Brazil

**Keywords:** hydroxide salts (HS), solid state synthesis, 2,3-dihydroperimidines, solvent-free, recuperation, paratacamite-like structures

## Abstract

Herein, we report the synthesis, characterization, and catalytic evaluation of cobalt hydroxide chloride [Co_2_(OH)_3_Cl] in the solvent-free synthesis of 2-substituted 2,3-dihydroperimidines. The presented method aligns with several green chemistry principles, offering operational simplicity, purification by recrystallization, no by-product formation, high yields (64–99%), and short reaction times. A total of 16 dihydroperimidines were synthesized to demonstrate substrate scope versatility. Additionally, the catalyst was successfully recycled and reused in multiple cycles without significant loss. Its robustness was further confirmed by gram-scale synthesis, achieving an 89% yield.

## 1. Introduction

Cobalt-based hydroxychlorides are an important class of inorganic materials that have attracted significant interest due to their unique structural features and various technological uses [1]. Among these, cobalt hydroxychloride Co_2_(OH)_3_Cl is particularly notable for its structural resemblance to minerals of the atacamite group, such as paratacamite [Cu_3_(Cu,M)Cl_2_(OH)_6_, where M = Zn, Mg, Ni] and their synthetic analogues [2,3].

The atacamite group is characterized by complex three-dimensional framework structures made of interconnected metal octahedra [2,4]. These structures feature sheets of edge-sharing MCl_2_(OH)_4_ octahedra connected through interlayer metal sites coordinated by six OH^−^ ligands, forming M-OH-M bridges that create a strong three-dimensional network rather than a true layered structure [2,5]. The structural similarity between Co_2_(OH)_3_Cl and paratacamite-type compounds suggests that cobalt hydroxychloride likely adopts a similar three-dimensional framework, with Co^2+^ ions occupying both the sheet-forming and interlayer octahedral sites.

Metal hydroxysalts, whether layered or framework-structured, have found applications in environmental sustainability [6], energy storage [7], water treatment [8], drug delivery [9,10], photocatalysis [11,12] and catalysis [12,13] due to their unique properties such as layered structure, memory effect, selective ion exchange, high surface area, tuneable bandgap, and the presence of Brønsted–Lowry and Lewis acid/base sites [6,14,15]. Furthermore, these materials offer advantages such as high stability, low cost, low toxicity, facile synthesis, and reusability [6].

Among layered metal hydroxy salts, layered double hydroxides (LDHs) are well-established catalysts for diverse organic transformations. These include the synthesis of xanthenes, 1,4-dihydropyrimidines [16], fused pyrimidines [17], *β*-nitroalcohols [18], 2-aryl benzimidazoles, benzothiazoles, benzoxazoles [19], and oxidative amidation reactions [20]. In contrast, the catalytic applications of layered hydroxy salts (LHSs) in organic synthesis remain scarce, limited primarily to biodiesel esterification [21,22] and click reactions for synthesizing 1,2,3-triazoles [23]. Notably, the use of specific metal hydroxysalts, such as Co_2_(OH)_3_Cl, is, to our knowledge, absent from the literature.

Perimidines are a class of *N*-heterocyclic compounds with notable pharmacological potential, exhibiting antioxidants [24], antimicrobial [25,26], anticancer [27,28] and anti-inflammatory properties [29,30]. Their synthesis usually involves a condensation reaction between 1,8-diaminonaphthalene and aldehydes, often catalyzed by Brønsted–Lowry [31] or Lewis’s acid catalysts [32,33].

In this context, cobalt-based catalysts have demonstrated efficiency in organic synthesis [34,35,36,37], and some have been explored for perimidines synthesis. However, existing methods frequently require calcination [38] or ligand complexation followed by pyrolysis [39], highlighting the need for simpler, more sustainable catalytic systems. Therefore, the screening for simpler cobalt catalysts remains an area of interest. Furthermore, in recent years, cobalt-catalyzed solvent-free reactions have gained significant attention in the scientific community due to their alignment with the principles of sustainable chemistry [40,41,42,43].

To the best of our knowledge, the application of cobalt as a catalyst in the synthesis of 2-substituted 2,3-dihydroperimidines via condensation reaction has not yet been explored. Thus, in connection with our continuing interest in designing new synthetic methodologies, transition metal-catalysis, as well as biologically active *N*-heterocyclic compounds are explored [44,45,46,47,48,49]; herein, we present a comprehensive investigation of Co_2_(OH)_3_Cl synthesis via urea-mediated hydrolysis, focusing on the structural characterization, phase identification, and property evaluation of the resulting material as a potential catalyst for 2,3-dihydro-1*H*-peridimines condensation reaction. Through detailed X-ray diffraction analysis, we establish the structural relationship between Co_2_(OH)_3_Cl and the paratacamite family, providing insights into the 3D framework that underpins its unique properties.

## 2. Results and Discussion

### 2.1. Cobalt Hydroxychloride [Co_2_(OH)_3_Cl] Characterization

#### 2.1.1. Structural and Phase Formation Characterization

The material’s structure was determined using the crystallographic chart COD2310848, corresponding to a hydroxy salt with the general formula Co_2_(OH)_3_Cl. It crystallizes in a hexagonal system (space group R-3m), with unit cell parameters a = b = 6.84 Å, c = 14.50 Å, and angles α = β = 90°, γ = 120°.

**Figure 1:**
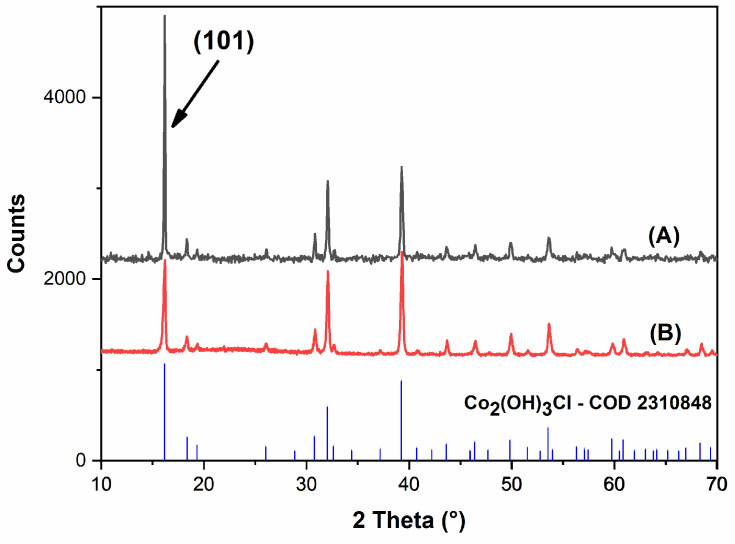
Diffractogram of Co_2_(OH)_3_Cl (A) as prepared (B) after application.

The XRD pattern in Figure 1A shows an intense reflection at the (101) plane at 16° (2θ), deviating from the typical (00l) pattern of classical brucite-like layered hydroxide salts (LHS), instead suggesting a paratacamite-type structure. This unique diffraction arises from monoatomic layers linked by octahedra, featuring 25% vacancies at alternating cobalt ion sites. These cations move to interlayer positions and are accompanied by a diagonal shift (a/√3) along the (120), which enhances the {101} planes [50].

Paratacamite is a polymorph of atacamite, and both display intense (101) reflections. However, they are distinguishable by their crystal symmetry: atacamite-like structures crystallize in an orthorhombic system (Pnma), which cannot transform into a hexagonal framework [51]. Conversely, as previously noted, paratacamite adopts a rhombohedral lattice (R-3m), compatible with the hexagonal system, as observed in the Co_2_(OH)_3_Cl material [51]. This structural arrangement further confirms that Cl^−^ ions are incorporated within the crystal lattice rather than in the interlayer region, distinguishing it from traditional layered hydroxy salt [50].

Furthermore, the XRD pattern shown in Figure 1B (after catalytic use) demonstrates that the crystalline framework remains largely intact after the perimidine condensation reaction. However, notable changes in the (101) peak profile, including both reduced intensity and increased peak broadening (full width at half maximum (FWHM), increasing from 0.124° to 0.301°), indicate structural modifications within the material. While several factors could contribute to these observations (including preferential orientation effects, surface modifications, adsorption of organic molecules, or localized disorder at interlayer sites), the (101) plane’s sensitivity to interlayer arrangements suggests that structural perturbations may have occurred at Co–OH–Co bridging regions. The combination of intensity reduction and peak broadening is consistent with the introduction of structural irregularities at these interlayer sites, which represent the most accessible regions for organic molecule interactions during catalysis.

#### 2.1.2. Vibrational Spectroscopy Analysis

**Figure 2:**
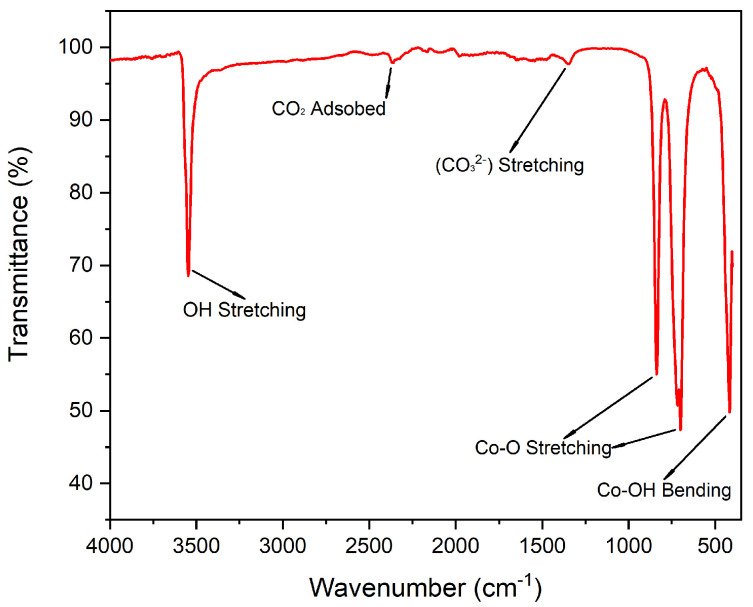
Co_2_(OH)_3_Cl ATR-FTIR spectrum.

Attenuated total reflectance Fourier transform infrared spectroscopy (ATR-FTIR) analysis (Figure 2) reveals a sharp absorption band at 3550 cm^−1^ corresponding to the stretching vibration of non-hydrogen-bonded OH, characteristic of brucite-like layered material [52,53]. The bands observed at 838 and 697 cm^−1^ are assigned to Co-O stretching vibrations, and the signal at 416 cm^−1^ is attributed to the bending mode of Co-OH bonds, respectively [53,54].

A minor absorption band at 2369 cm^−1^ suggests the presence of adsorbed atmospheric CO_2_ [53], and the band at 1350 cm^−1^ may be related to residual urea from the synthesis, usually indicating the presence of carbonate species (CO_3_^2−^) [52,55] interacting or incorporated into the LHS structure. Notably, no signals corresponding to H_2_O bound to the material were detected, suggesting enhanced availability of Brønsted–Lowry acidic and basic active sites due to the absence of competing water interactions.

#### 2.1.3. Electronic Behaviour

**Figure 3:**
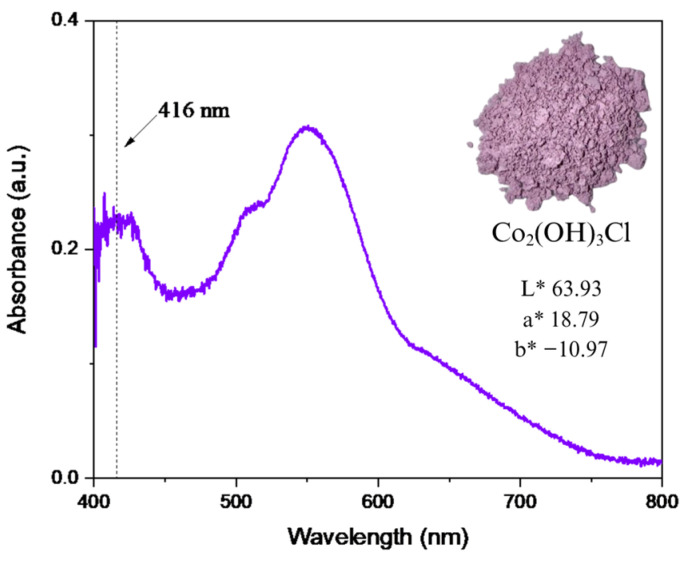
Electronic spectrum of Co_2_(OH)_3_Cl.

The diffuse reflectance UV-Vis spectrum (Figure 3) exhibits an absorption band at 416 nm, attributed to a ligand-to-metal charge transfer (LMCT) from O^2−^ (hydroxyl groups) to Co^2+^, consistent with observations in related transition metal LDH systems, where bands below 480 nm typically indicate surface octahedral M–OH sites [56]. In addition, the bands between 510 and 548 nm are assigned to the ^4^T_1g_ → ^4^T_1g_(P) and ^4^A_2g_(F) transitions of octahedral coordinated Co^2+^ (O_h_) [54,55]. Moreover, the absence of a band around 700 nm confirms that no Co^3+^ is present on octahedral sites [56,57,58], and similarly, the lack of bands near 650 nm indicates that Co^2+^ in tetrahedral sites is not present in the structure [55,56,57].

The material’s pink coloration is characteristic of β-Co(OH)_2_ brucite-like octahedral systems. Colorimetric analysis (CIE L*a*b*) yielded values of L* = 63.93, a* = 18.79, and b* = −10.97, with a chroma (C = 21.76)* and hue angle (h = 329.72°)* further defining its optical properties [58,59].

#### 2.1.4. Morphological, Surface, and Chemical Analysis

**Figure 4:**
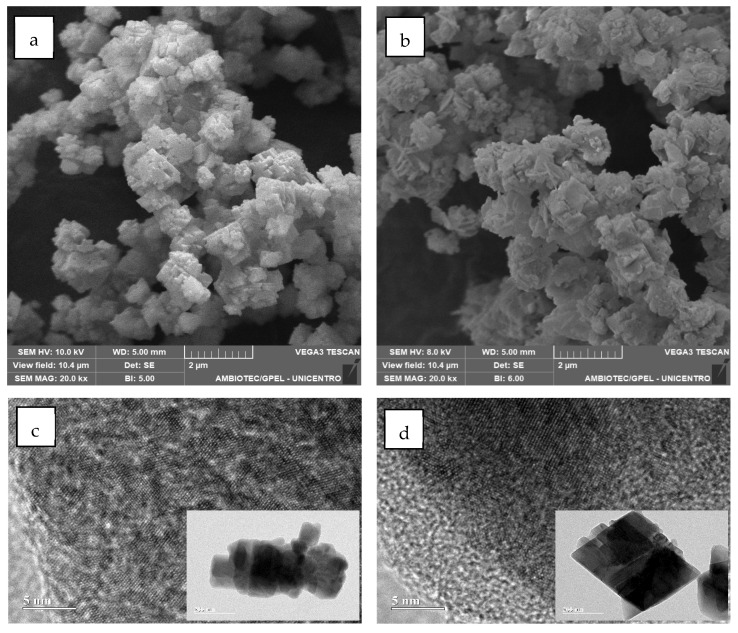
Scanning electron microscopy (SEM), and transmission electron microscopy (TEM) of Co_2_(OH)_3_Cl, before (**a**,**c**), and after the catalytic process (**b**,**d**). Scale bars: 2 μm (SEM) and 5 nm (TEM main images) and 200 nm (TEM insets).

SEM micrographs presented in Figure 4 reveal morphological changes in the Co_2_(OH)_3_Cl samples before (a) and after (b) perimidine catalysis. The post-catalytic sample exhibits a notable fragmentation pattern, with the original agglomerates breaking down into smaller plate-shaped particles, suggesting mechanical stress or structural reorganization during the catalytic process.

Complementary TEM analysis (Figure 4c,d) confirms the preservation of crystalline order at the nanoscale level, with well-defined atomic plane coherence maintained throughout the material. The inset images showing individual crystallites demonstrate that despite the morphological fragmentation observed by SEM, the particles retain their crystalline structure with well-organized lattice fringes, indicating that the structural modifications are localized rather than involving complete crystalline breakdown.

This behaviour is beneficial for catalyst use because it provides more contact area, which offers more active sites. Conversely, the results from the B.E.T. analysis on the surface area and porosity of the material show that Co_2_(OH)_3_Cl has a low surface area of 3.717 m^2^/g. Regarding porosity, it has a total pore volume of 0.0056 cm^3^/g and a pore size of 1.809 nm, classified as microporous based on IUPAC standards. Elemental analysis by EDS showed 55% Co, 28% O, and 17% Cl, aligning with the composition of Co_2_(OH)_3_Cl with a paratacamite-type structure [50].

### 2.2. Synthesis of 2-Substituted 2,3-dihydro-1H-perimidines

The model reaction for optimizing reaction conditions was conducted between 1,8-diaminonaphthalene (**1**) and benzaldehyde (**2a**), with variations in catalyst type, temperature, and catalyst loading (Table 1). Initially, a study was carried out to determine the best catalyst, from a variety of cobalt hydroxide salts, to be used in the reaction for the synthesis of perimidine **3a**, under solvent-free conditions, 1 mol% of catalyst, at 70 °C for 5 min (Table 1, entries 1–3). Thus, it was observed that the counter ion used in the salt had a significant influence on the reaction yield, since when sulphate and nitrate were used, the product **3a** was obtained in 67 and 86% yield (Table 1, entries 1 and 2), respectively, while when chloride [Co_2_(OH)_3_Cl] was used the product was obtained in 96% yield (Table 1, entry 3). The influence of temperature was highly significant. Lowering the temperature to 60 °C drastically reduced the yield of **3a** to 30% (Table 1, entry 4). This is likely due to the partial fusion of 1,8-diaminonaphthalene, which has a reported melting point of 65 °C, hindering reactant mobility. Finally, an increase in catalyst load did not have a significant influence on product yield (Table 1, entry 5).

With optimized reaction conditions in hand, next we explored the generality and scope of this catalytic system. For this purpose, a wide range of aldehydes was employed, totaling 16 examples to evaluate the scope of the synthetic route, as presented in Table 2.

The reaction of unsubstituted benzaldehyde (**2a**) afforded the desired perimidine product (**3a**) in 96% yield (Table 2, entry 1). The introduction of methyl substituents showed interesting effects: *ortho*-tolualdehyde (**2b**) gave a quantitative yield (99%) of perimidine (**3b**), while *para*-tolualdehyde (**2c**) provided a slightly lower yield of 84% (Table 2, entries 2–3). Notably, cinnamaldehyde (**2d**), featuring a vinylic group attached to the aromatic ring, proved to be an excellent substrate, yielding perimidine (**3d**) in 97% (Table 2, entry 4). The catalyst system showed good compatibility with halogenated substrates. Bromo-substituted aldehydes at both *meta-* (**2e**) and *para-* (**2f**) positions gave the corresponding perimidines (**3e–3f**) in 88% and 84% yields, respectively (Table 2, entries 5–6). Similarly, chloro-substituted analogues at *ortho* (**2g**) and *para* (**2h**) positions afforded products (**3g–3h**) in comparable yields of 84% and 85% (Table 2, entries 7–8). Oxygen-containing substituents were particularly effective, with *para*-hydroxybenzaldehyde (**2i**) and *para*-anisaldehyde (**2j**) yielding perimidines (**3i–3j**) in excellent yields of 94% and 85%, respectively (Table 2, entries 9–10). The methodology extended successfully to heteroaromatic aldehydes. Pyridine-carboxaldehydes with varying nitrogen positions (*ortho* **2k**, *meta* **2l**, *para* **2m**) gave products (**3k–3m**) in 85%, 93%, and 88% yields (Table 2, entries 11–13). Other heterocyclic substrates, including thiophene-2-carboxaldehyde (**2n**) and furfural (**2o**), provided the expected perimidines (**3n–3o**), albeit with somewhat lower yields of 80% and 64% (Table 2, entries 14–15). The system also accommodated more complex substrates, as evidenced by the 88% yield obtained for the indole-containing perimidine (**3p**) (Table 2, entry 16).

The recyclability of the Co_2_(OH)_3_Cl catalyst was evaluated using the model reaction between 1,8-diaminonaphthalene (**1**) and benzaldehyde (**2a**) to produce perimidine (**3a**) (Table 3). Initial attempts to recover the catalyst by simple filtration proved ineffective due to the small mass of catalyst employed (1 mol%). To overcome this challenge, we implemented a centrifugation protocol prior to recrystallization, washing the organic phase with ethanol (3 × 5 mL) at 3000 rpm for 2 min. This approach successfully recovered the catalyst while maintaining good catalytic performance. The recycled catalyst demonstrated consistent efficiency for two cycles, yielding perimidine (**3a**) in approximately 90% yield for each run. However, a noticeable decrease in activity was observed in the third cycle, with the yield dropping to 71% (Table 3). This reduction in efficiency suggests potential catalyst degradation or loss during the recovery process.

**Figure 5:**
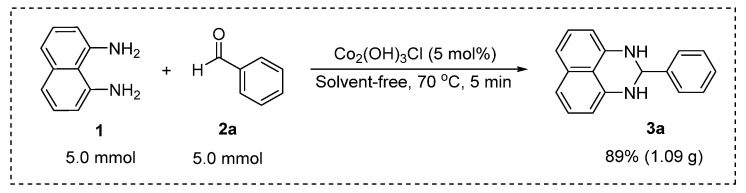
Gram-scale synthesis for perimidine **3a**.

To evaluate the practical applicability of this methodology, we performed a gram-scale synthesis using 1,8-diaminonaphthalene (**1**) (5.0 mmol, 791 mg) and benzaldehyde (**2a**) (5.0 mmol, 510 μL) in the presence of 5 mol% Co_2_(OH)_3_Cl catalyst (0.05 mmol, 51 mg). As illustrated in Figure 5, this scaled-up reaction successfully afforded perimidine **3a** in 89% yield (1.09 g), confirming the robustness and scalability of the catalytic system. The maintained high yield at increased scale underscores the method’s potential for practical synthetic applications.

**Figure 6:**
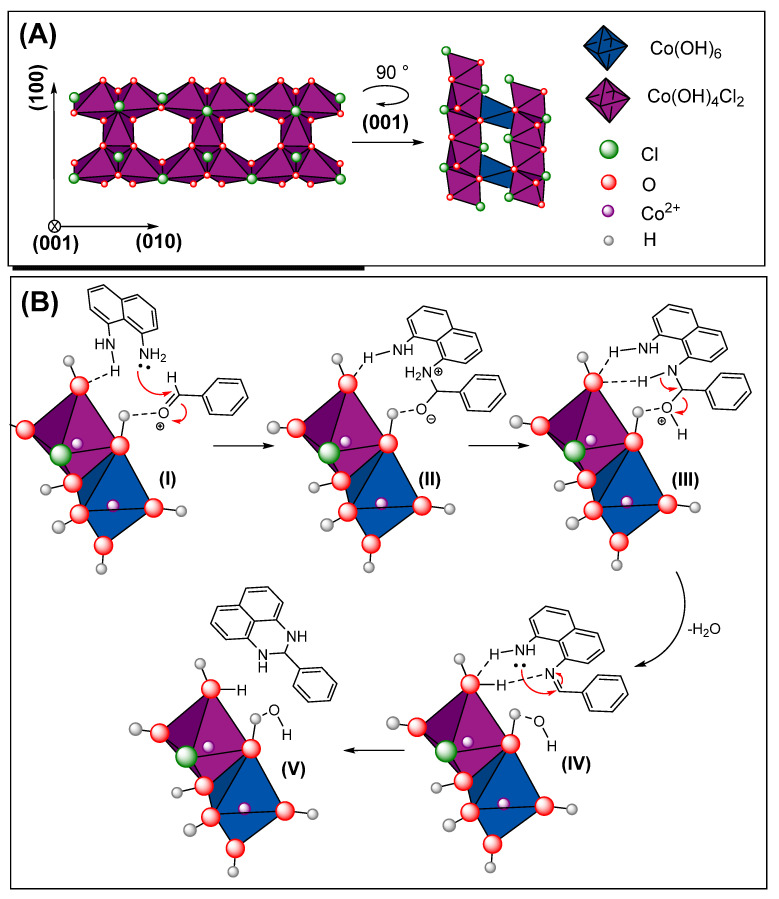
Co_2_(OH)_3_Cl compound structure (**A**) and proposed mechanism of catalytic effect in 2,3-dihydro-1*H*-perimidine condensation (**B**).

As previously discussed, condensation reactions with perimidine commonly employ acid catalysts [32,33]. In this context, both water [60] and glycerol [61] have been employed as reaction media for perimidine synthesis, where solvent hydroxyl groups facilitate aldehyde activation. Building on these observations, we propose that the surface hydroxyl groups of Co_2_(OH)_3_Cl serve as the primary catalytic sites in our system. These hydroxyl groups are also proposed as bifunctional centres, due to the amphoteric behaviour in hydroxylated oxides [62]. As illustrated in Figure 6, these sites likely coordinate with 1,8-diaminonaphthalene, thereby promoting nucleophilic addition to the activated aldehyde. Furthermore, surface Co^2+^ centres may stabilize the imine intermediate, consistent with previous reports on cobalt-based catalysts [63,64]. Supporting this mechanism, the UV-Vis spectrum reveals a ligand-to-metal charge transfer (LMCT) band at 416 nm, confirming the presence of strongly basic O^2−^ sites [65]. These sites are expected to preferentially coordinate with 1,8-diaminonaphthalene, enhancing reactant interaction and facilitating the condensation process.

The catalytic cycle begins with aldehyde activation through coordination to acidic hydroxyl sites on the Co_2_(OH)_3_Cl surface (**I**), followed by proton transfer from the nucleophilic amine to the carbonyl oxygen (**II**). The activated 1,8-diaminonaphthalene then attacks the electrophilic carbonyl carbon, forming a Schiff base intermediate with concomitant water elimination (**III**). T he liberated water molecule remains coordinated to the catalyst structure. The resulting imine intermediate is stabilized through two potential pathways: (a) coordination to surface Co^2+^ Lewis acid sites or (b) hydrogen bonding with surface hydroxyl groups, which generates a partial positive charge resembling an iminium ion (**IV**). This enhanced electrophilicity facilitates intramolecular cyclization via nucleophilic attack by the second amine group, ultimately yielding the perimidine product (**V**), Figure 6.

## 3. Materials and Methods

### 3.1. Reagents and Apparatus

#### 3.1.1. Reagents and Characterization for [Co_2_(OH)_3_Cl]

All reagents were commercially obtained to synthesize cobalt hydroxychloride [Co_2_(OH)_3_Cl]. The material’s structure and phase were studied using X-ray diffraction measurements performed on a Bruker diffractometer XRD-D2 Phaser, with a copper source (λ = 1.5418 Å), covering 3° to 70° (2θ), with 0.2°/s increments. Infrared spectra recorded in attenuated total reflectance (ATR) mode were obtained with a PerkinElmer Frontier spectrophotometer, in the 4000–650 cm^−1^ range. The optical diffuse absorbance was measured on the powder form of the compound using a Varian UV-VIS-NIR spectrophotometer CARYb5G in the 400–800 nm range. Colorimetric parameters were analyzed with a portable colorimeter (NR60CP-3nh), equipped with a D65 light source and an 8 mm measuring aperture. The surface area, pore size, and volume were determined through nitrogen adsorption analysis (B.E.T.), using an Anton Paar Nova 800 instrument. The sample was analyzed in a 9 mm cell and vacuum degassed for 4 h at 180 °C. Transmission Electron Microscope (TEM) images were obtained in a JEOL JEM-2100, Tokyo, Japan, equipped with an Energy-Dispersive X-ray Spectrometer (EDS).

#### 3.1.2. Reagents and Characterization for 2,3-Dihydro-1H-perimidines

The reagents 1,8-diaminonaphthalene, aldehydes, and solvents were commercially acquired. The Nuclear Magnetic Resonance (NMR) spectra were obtained on a Bruker AVANCE NEO-500 and Bruker DPX-300 spectrometers using CDCl_3_ as the solvent for Hydrogen and Carbon (^1^H 500 and 300 MHz and ^13^C 125 and 75 MHz NMR). All chemical shifts were reported in parts per million (ppm), with the ^1^H spectrum referenced to 0.00 ppm (TMS) and the ^13^C spectrum referenced to 77.16 ppm (CDCl_3_). The thin-layer chromatography (TLC) plates utilized were GF254 silica with a thickness of 0.20 mm, from the brand Macherey-Nagel, and the visualization methods used were iodine chamber and ultraviolet light (254 nm).

### 3.2. Experimental Procedures

#### 3.2.1. Cobalt Hydroxichloride [Co_2_(OH)_3_Cl] Solid State Synthesis

The cobalt hydroxichloride [Co_2_(OH)_3_Cl] was produced through a solid-state reaction. The decomposition of urea is necessary to supply the hydroxide ions for synthesis, as explained by Rajamathi (2001) [59]. Therefore, 9.0 g of cobalt (II) chloride hexahydrate (CoCl_2_·6H_2_O) and 2.0 g of urea were dissolved in 2 mL of distilled water in a hermetically sealed reaction flask, then heated at 110 °C for four hours in an oven. After cooling to room temperature, the resulting material was washed with distilled water and centrifuged five times to remove impurities. The solid was then dried in an oven at 40 °C for five days. Afterwards, the sample was characterized using XRD, FTIR, TEM/EDS, and UV-Vis.

#### 3.2.2. Synthesis of 2-Substituted 2,3-dihydro-1H-permidine

In a 5 mL test tube, 79.1 mg (0.5 mmol) of 1,8-diaminonaphthalene **1** and Co_2_(OH)_3_Cl (1 mol%, 1.0 mg) were added, heated until 70 °C, and left under stirring until the reaction mixture had fused. Subsequently, the respective aldehyde **2a-p** (0.5 mmol) was added. The reaction progress was monitored by TLC until complete consumption of the starting materials.

Following the reaction completion, the organic phase was solubilized in ethanol (10 mL) and filtered to separate the Co_2_(OH)_3_Cl catalyst. The product in the organic phase was then recrystallized in a cold-water bath (15 mL). Finally, the precipitate was filtered and dried at an ambient temperature. The described spectra data are presented below, and the spectra are found in the Supplementary Materials (Figures S1–S32).

**2-phenyl-2,3-dihydro-1*H*-perimidine (3a)** [66]: 118 mg (96%), ^1^H NMR (300 MHz, CDCl_3_) δ 7.66–7.27 (m, 9H), 6.54 (d, *J* = 6.1 Hz, 2H), 5.47 (s, 1H), 4.54 (s, 1H). ^13^C NMR (75 MHz, CDCl_3_) δ 142.23, 140.21, 135.02, 129.72, 128.96, 128.03, 127.00, 117.99, 113.57, 105.95, 68.50.

**2-(o-tolyl)-2,3-dihydro-1*H*-perimidine (3b)** [66]: 129 mg (99%), ^1^H NMR (300 MHz, CDCl_3_) δ 7.74(s, 1H), 7.23 (m, 7H), 6.51 (s, 2H), 5.71 (s, 1H), 4.43 (s, 2H), 2.50 (s, 3H). ^13^C NMR (75 MHz, CDCl_3_) δ 142.54, 137.66, 136.66, 135.11, 131.06, 129.20, 128.21, 126.96, 126.76, 117.97, 113.71, 106.09, 65.27, 19.25.

**2-(p-tolyl)-2,3-dihydro-1*H*-perimidine (3c)** [66]: 109 mg (84%), ^1^H NMR (300 MHz, C_6_D_6_) δ 7.50 (d, *J* = 6.9, 2H), 7.24–7.22 (m, 6H), 6.49 (d, *J* = 6.3, 2H), 5.41 (s, 1H), 4.49 (s, 2H), 2.39 (s, 3H). ^13^C NMR (75 MHz, CDCl_3_) δ 142.35, 139.61, 137.34, 135.04, 129.59, 127.90, 126.99, 117.92, 113.59, 105.90, 68.31, 21.41.

**(E)-2-styryl-2,3-dihydro-1*H-*perimidine (3d**) [67]: 132 mg (97%), ^1^H NMR (300 MHz, CDCl_3_) δ 7.35–7.19 (m, 9H), 6.69 (d, *J* = 15.7 Hz, 1H), 6.48 (d, *J* = 7.3, 2H), 6.34 (dd, *J* = 15.6, 7.6 Hz, 1H), 5.00 (d, *J* = 7.3, 2H), 4.42 (s, 2H). ^13^C NMR (75 MHz, CDCl_3_) δ 141.19, 135.74, 134.86, 134.67, 128.81, 128.58, 128.06, 127.61, 127.02, 117.87, 113.54, 106.16, 67.08.

**2-(3-bromophenyl)-2,3-dihydro-1*H*-perimidine (3e)** [56]: 142 mg (88%), ^1^H NMR (300 MHz, CDCl_3_) δ 7.82 (s, 1H), 7.55 (s, 1H), 7.25 (m, 6H), 6.54 (s, 2H), 5.42 (s, 1H), 4.43 (s, 2H). ^13^C NMR (75 MHz, CDCl_3_) δ 142.48, 141.78, 134.97, 132.82, 131.27, 130.53, 127.03, 126.70, 122.97, 118.29, 113.52, 106.17, 67.80.

**2-(4-bromophenyl)-2,3-dihydro-1*H*-perimidine (3f)** [66]: 136 mg (84%), ^1^H NMR (500 MHz, CDCl_3_) δ 7.52 (s, 2H), 7.44 (s, 2H), 7.21 (m, 4H), 6.48 (s, 2H), 5.34 (s, 1H), 4.42 (s, 2H). ^13^C NMR (126 MHz, CDCl_3_) δ 141.84, 139.21, 134.93, 132.07, 129.71, 127.00, 123.66, 118.19, 113.47, 106.08, 67.78.

**2-(2-chlorophenyl)-2,3-dihydro-1*H*-perimidine (3g)** [66]: 118 mg (84%), ^1^H NMR (300 MHz, CDCl_3_) δ 7.82 (s, 1H), 7,41–7.24 (m, 7H), 6.57 (s, 2H), 5.98 (s, 1H), 4.60 (s, 2H). ^13^C NMR (75 MHz, CDCl_3_) δ 141.69, 137.78, 134.95, 133.33, 130.29, 129.84, 129.17, 127.77, 127.06, 118.16, 113.42, 106.24, 63.94.

**2-(4-chlorophenyl)-2,3-dihydro-1*H*-perimidine (3h)** [66]: 119 mg (85%),^1^H NMR (300 MHz, CDCl_3_) δ 7.54 (d, *J* = 8.2 Hz, 1H), 7.40 (d, *J* = 8.2 Hz, 1H), 7.28–7.23 (m, 4H), 6.50 (d, *J* = 6.6 Hz, 2H), 5.40 (s, 1h), 4.45 (s, 2H). ^13^C NMR (75 MHz, CDCl_3_) δ 141.91, 138.74, 135.50, 134.96, 129.44, 129.15, 127.01, 118.21, 113.51, 106.09, 67.78.

**4-(2,3-dihydro-1*H*-perimidin-2-yl)phenol (3i)** [67]: 123 mg (94%), ^1^H NMR (300 MHz, CDCl_3_) δ 7.50 (d, *J* = 7.9 Hz, 1H), 7.26–7.23 (m, 4H), 6.88 (d, *J* = 7.9 Hz, 2H), 6.52 (d, *J* = 6.4 Hz, 2H), 5.40 (s, 1H), 4.49 (s, 2H). ^13^C NMR (75 MHz, CDCl_3_) δ 156.96, 142.41, 135.07, 132.42, 129.51, 127.02, 117.99, 115.73, 113.62, 105.93, 68.11.

**2-(4-methoxyphenyl)-2,3-dihydro-1*H*-perimidine (3j)** [66]: 117 mg (85%), ^1^H NMR (300 MHz, CDCl_3_) δ 7.54 (d, *J* = 8.4 Hz, 2H), 7.27–7.18 (m, 4H), 6.94 (d, *J* = 8.4 Hz, 2H), 6.49 (d, *J* = 6.3 Hz, 2H) 5.39 (s, 1H), 4.48 (s, 2H), 3.83 (s, 3H). ^13^C NMR (75 MHz, CDCl_3_) δ 160.67, 142.42, 135.05, 132.43, 129.28, 127.00, 117.91, 114.24, 113.57, 105.85, 68.06, 55.52.

**2-(pyridin-2-yl)-2,3-dihydro-1*H*-perimidine (3k)** [68]: 105 mg (85%), ^1^H NMR (500 MHz, CDCl_3_) δ 8.54 (d, *J* = 4.8 Hz, 1H), 7.58 (td, *J* = 7.7, 1.4 Hz, 1H)., 7.50 (d, *J* = 7.9 Hz, 1H), 7.23–7.15 (m, 5H), 6.53 (d, *J* = 7.1 Hz, 2H), 5.51 (s, 1H), 4.98 (s, 2H). ^13^C NMR (126 MHz, CDCl_3_) δ 159.30, 149.27, 137.25, 134.75, 126.96, 123.59, 120.80, 118.00, 113.96, 106.62, 67.65.

**3-(pyridin-2-yl)-2,3-dihydro-1*H*-perimidine (3l)**: 115 mg (93%), ^1^H NMR (500 MHz, CDCl_3_) δ 8.70 (s, 1H), 8.62 (d, *J* = 4.0 Hz, 1H), 7.95 (dd, *J* = 9.7, 1.8 Hz, 1H), 7.33 (dd, *J* = 7.8, 4.9 Hz, 1H), 7.24–7.21 (m, 4H), 6.51 (dd, *J* = 6.6, 1.6 Hz, 2H), 5.42 (s, 1H), 4.58 (s, 2H). ^13^C NMR (126 MHz, CDCl_3_) δ 150.94, 149.49, 141.68, 135.97, 134.87, 126.99, 124.03, 118.53, 118.32, 113.44, 106.20, 66.13.

**4-(pyridin-2-yl)-2,3-dihydro-1*H*-perimidine (3m)**: 109 mg (88%), ^1^H NMR (500 MHz, CDCl_3_) δ 8.69 (d, *J* = 5.9 Hz, 1H), 7.55 (d, *J* = 5.9 Hz, 1H), 7.28–7.25 (m, 3H), 6.57 (dd, *J* = 6.4, 1.8 Hz, 2H), 5.48 (s 1H), 4.53 (s, 2H). ^13^C NMR (126 MHz, CDCl_3_) δ 150.66, 148.89, 141.19, 134.92, 127.07, 122.72, 118.60, 113.62, 106.52, 67.19.

**2-(thiophen-2-yl)-2,3-dihydro-1*H*-perimidine (3n)** [68,69]: 101 mg (80%),^1^H NMR (500 MHz, CDCl_3_) δ 7.38 (s, 1H), 7.25–7.23 (m, 5H), 7.02 (s, 1H), 6.53 (s, 2H), 5.80 (s, 1H), 4.65 (s, 2H). ^13^C NMR (126 MHz, CDCl_3_) δ 144.15, 141.47, 134.94, 127.24, 127.02, 126.56, 126.45, 118.33, 113.82, 106.29, 63.90.

**2-(furan-2-yl)-2,3-dihydro-1*H*-perimidine (3o)** [70,71]: 76 mg (64%),^1^H NMR (500 MHz, CDCl_3_) δ 7.42 (s, 1H), 7.26–7.22 (m, 4H), 6.59 (d, *J* = 6.6 Hz, 2H), 6.42 (s, 1H), 6.36 (s, 1H), 5.65 (s, 1H), 4.70 (s, 2H). ^13^C NMR (126 MHz, CDCl_3_) δ 153.54, 142.81, 140.83, 134.88, 127.01, 118.44, 114.04, 110.66, 107.79, 106.79, 61.68.

**3-(1H-indol-3-yl)-2,3-dihydro-1*H*-perimidine (3p)**: 125 mg (88%), ^1^H NMR (500 MHz, CDCl_3_) δ 8.25 (s, 1H), 7.90 (d, *J* = 7.9 Hz, 1H), 7.32 (d, *J* = 8.2 Hz, 1H), 7.26–7.08 (m, 7H), 6.47 (d, *J* = 7.9 Hz, 1H), 5.79 (s, 1H), 4.60 (s, 2H). ^13^C NMR (126 MHz, CDCl_3_) δ 142.78, 136.32, 135.06, 127.02, 125.62, 123.76, 122.64, 120.07, 119.89, 117.69, 115.11, 113.85, 111.61, 105.91, 62.17.

#### 3.2.3. Co_2_(OH)_3_Cl Recycling

In a 50 mL round-bottom flask, 1,8-diaminonaphthalene **1** (0.5 mmol) and 1 mol% of catalyst (1.0 mg) were added. The mixture was melted while stirring, and then benzaldehyde **2a** (0.5 mmol) was added. TLC confirmed the reaction finished after 5 min. The crude product was then dissolved in ethanol (5 mL), transferred to a Falcon tube, centrifuged for 2 min at 3000 rpm, and the catalyst was dried either in an oven at 40 °C for 2 h or under vacuum using a rotary evaporator. The organic phase was recrystallized with water (20 mL) in an ice bath, and this process was repeated three times.

#### 3.2.4. Scale-Up Synthesis

In a 50 mL round-bottom flask, a magnetic stir bar was added, followed by 1,8-diaminonaphthalene 1 (5.0 mmol, 792 mg) and Co_2_(OH)_3_Cl (5 mol%, 51 mg). Then, after the medium was melted and under stirring, benzaldehyde **2a** (5.0 mmol, 510 µL) was added.

Afterwards, after 5 min, the reaction was complete, and the round-bottom flask was removed from the temperature; completion was confirmed through TLC. The crude product was dissolved in 10 mL of ethanol, filtered to remove the catalyst, and finally recrystallized in 15 mL of cold water to produce product **3a**.

## 4. Conclusions

A facile solid-state synthesis was developed for the preparation of the layered hydroxy salt Co_2_(OH)_3_Cl, utilizing urea and minimal water (2 mL). Comprehensive characterization by XRD, FTIR, UV-Vis spectroscopy, and SEM/EDS confirmed the formation of a paratacamite-type structure with well-defined crystallinity and morphology. The catalytic potential of Co_2_(OH)_3_Cl was successfully demonstrated in the efficient synthesis of perimidines, which are pharmaceutically relevant heterocycles. Under optimized conditions (70 °C, solvent-free, 1 mol% catalyst, 5 min), the reaction exhibited a broad substrate scope, accommodating 16 diverse aryl aldehydes with yields ranging from 64% to 99%. Notably, the catalyst retained high activity (~90% yield) over two consecutive cycles, confirming its reusability and structural stability. Furthermore, the methodology proved scalable, delivering 1.09 g (89% yield) of perimidine in a gram-scale reaction, highlighting its potential for practical applications.

This work presents Co_2_(OH)_3_Cl as a sustainable, efficient, and reusable catalyst for the rapid synthesis of perimidines under mild conditions, offering a promising alternative to conventional acid-catalyzed approaches.

## Figures and Tables

**Table 1:** Reaction parameters optimization ^a^.

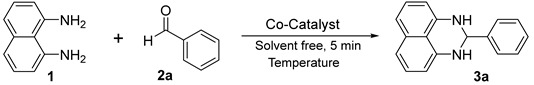			
Entry	Catalyst (mol%)	T (°C)	Yield (%) ^b^
1	Co_2_(OH)_3_SO_4_ (1)	70	67
2	Co_2_(OH)_3_NO_3_ (1)	70	86
3	Co_2_(OH)_3_Cl (1)	70	96
4	Co_2_(OH)_3_Cl (1)	60	30
5	Co_2_(OH)_3_Cl (5)	70	94

^a^ Reaction conditions: **1** (1 mmol), **2a** (1 mmol), catalyst, temperature, 5 min. ^b^ Isolated yield.

**Table 2:** Synthesis of Co_2_(OH)_3_Cl-catalyzed perimidines **3a-p** ^a^.

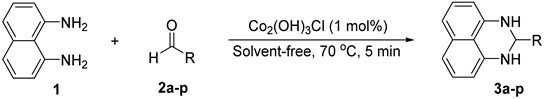			
Entry	Aldehyde	Product	Yield (%) ^b^
1	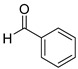 **2a**	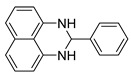 **3a**	96
2	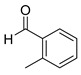 **2b**	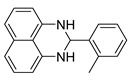 **3b**	99
3	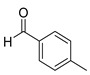 **2c**	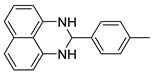 **3c**	84
4	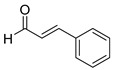 **2d**	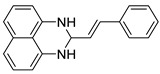 **3d**	97
5	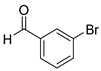 **2e**	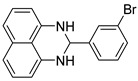 **3e**	88
6	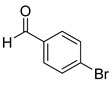 **2f**	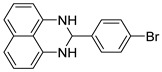 **3f**	84
7	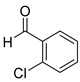 **2g**	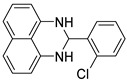 **3g**	84
8	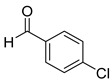 **2h**	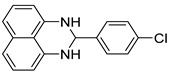 **3h**	85
9	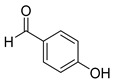 **2i**	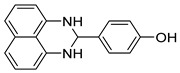 **3i**	94
10	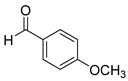 **2j**	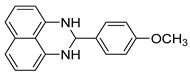 **3j**	85
11	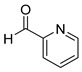 **2k**	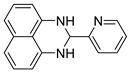 **3k**	85
12	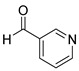 **2l**	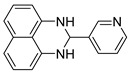 **3l**	93
13	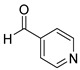 **2m**	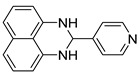 **3m**	88
14	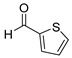 **2n**	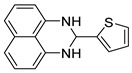 **3n**	80
15	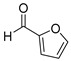 **2o**	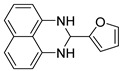 **3o**	64
16	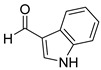 **2p**	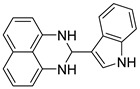 **3p**	88

^a^ Reaction conditions: **1** (0.5 mmol), aldehyde (0.5 mmol), Co_2_(OH)_3_Cl (1 mol%), 70 °C, 5 min. ^b^ Isolated yield.

**Table 3:** Catalyst recycling ^a^.

Entry	Cycle	Yield (%) ^b^
1	1°	96
2	2°	90
3	3°	71

^a^ Reactions conditions: **1** (1 mmol), **2a** (1 mmol), Co_2_(OH)_3_Cl (1 mol%), 70 °C, 5 min. ^b^ Isolated yield.

## Data Availability

Data are provided in the article.

## Supplementary Materials

The following supporting information can be downloaded at https://www.mdpi.com/article/10.3390/molecules31010182/s1. NMR Spectra (Figures S1–S32).
