# Supplementary material for: Synthesis of Cobalt Hydroxychloride and Its Application as a Catalyst in the Condensation of Perimidines
Source: Molecules. 2026 Jan 4;31(1):182. doi: 10.3390/molecules31010182 (PMC12787797; doi:10.3390/molecules31010182)
Supplement: Supplementary file 1 [file molecules-31-00182-s001.zip › molecules-3851015-supplementary.pdf]

Supplementary Materials

Synthesis of cobalt hydroxychloride and its application as catalyst in perimidines condensation

Cássio Siqueira [1,‡], Gabriela R.Borges [2,‡], Fernanda S. Portela [1], Maria E. Miks [1], Felipe F. Marques [3], Gleison A. Casagrande [2], Sumbal Saba [3,*], Rafael Marangoni [1,*], Jamal Rafique [2,3,*], Giancarlo V. Botteselle [1]

[1] Laboratory of Organic Synthesis and Catalysis (LabSOC), Midwestern Parana State University - UNICENTRO, Guarapuava, Paraná 85040-167, Brazil

[2] Instituto de Química (INQUI), Universidade Federal do Mato Grosso do Sul - UFMS, Campo Grande, 79074-460, MS – Brazil.

[3] Laboratory of Sustainable Synthesis and Organochalcogen (LabSO), Instituto de Química (IQ), Universidade Federal de Goiás – UFG, Goiânia, 74690-900, GO – Brazil.

‡ Both authors contributed equally to this work.

* Correspondence: sumbalsaba@ufg.br (S.S.); rmarangoni@unicentro.br (R.M.);
jamal.rafique@ufms.br , jamal.chm@gmail.com (J.R.)

Table of Contents:

Figure S1. ^{1}H NMR spectrum (300 MHz, $CDCl_3$) of compound **3a**
Figure S2. ^{13}C NMR spectrum (75 MHz, $CDCl_3$) of compound **3a**
Figure S3. ^{1}H NMR spectrum (300 MHz, $CDCl_3$) of compound **3b**
Figure S4. ^{13}C NMR spectrum (75 MHz, $CDCl_3$) of compound **3b**
Figure S5. ^{1}H NMR spectrum (300 MHz, $CDCl_3$) of compound **3c**
Figure S6. ^{13}C NMR spectrum (75 MHz, $CDCl_3$) of compound **3c**
Figure S7. ^{1}H NMR spectrum (300 MHz, $CDCl_3$) of compound **3d**
Figure S8. ^{13}C NMR spectrum (75 MHz, $CDCl_3$) of compound **3d**
Figure S9. ^{1}H NMR spectrum (300 MHz, $CDCl_3$) of compound **3e**
Figure S10. ^{13}C NMR spectrum (75 MHz, $CDCl_3$) of compound **3e**
Figure S11. ^{1}H NMR spectrum (500 MHz, $CDCl_3$) of compound **3f**
Figure S12. ^{13}C NMR spectrum (126 MHz, $CDCl_3$) of compound **3f**
Figure S13. ^{1}H NMR spectrum (300 MHz, $CDCl_3$) of compound **3g**
Figure S14. ^{13}C NMR spectrum (75 MHz, $CDCl_3$) of compound **3g**
Figure S15. ^{1}H NMR spectrum (300 MHz, $CDCl_3$) of compound **3h**
Figure S16. ^{13}C NMR spectrum (75 MHz, $CDCl_3$) of compound **3h**
Figure S17. ^{1}H NMR spectrum (300 MHz, $CDCl_3$) of compound **3i**
Figure S18. ^{13}C NMR spectrum (75 MHz, $CDCl_3$) of compound **3i**
Figure S19. ^{1}H NMR spectrum (300 MHz, $CDCl_3$) of compound **3j**
Figure S20. ^{13}C NMR spectrum (75 MHz, $CDCl_3$) of compound **3j**
Figure S21. ^{1}H NMR spectrum (500 MHz, $CDCl_3$) of compound **3k**
Figure S22. ^{13}C NMR spectrum (126 MHz, $CDCl_3$) of compound **3k**
Figure S23. ^{1}H NMR spectrum (500 MHz, $CDCl_3$) of compound **3l**
Figure S24. ^{13}C NMR spectrum (126 MHz, $CDCl_3$) of compound **3l**
Figure S25. ^{1}H NMR spectrum (500 MHz, $CDCl_3$) of compound **3m**
Figure S26. ^{13}C NMR spectrum (126 MHz, $CDCl_3$) of compound **3m**
Figure S27. ^{1}H NMR spectrum (500 MHz, $CDCl_3$) of compound **3n**
Figure S28. ^{13}C NMR spectrum (126 MHz, $CDCl_3$) of compound **3n**
Figure S29. ^{1}H NMR spectrum (500 MHz, $CDCl_3$) of compound **3o**
Figure S30. ^{13}C NMR spectrum (126 MHz, $CDCl_3$) of compound **3o**
Figure S31. ^{1}H NMR spectrum (500 MHz, $CDCl_3$) of compound **3p**
Figure S32. ^{13}C NMR spectrum (126 MHz, $CDCl_3$) of compound **3p**

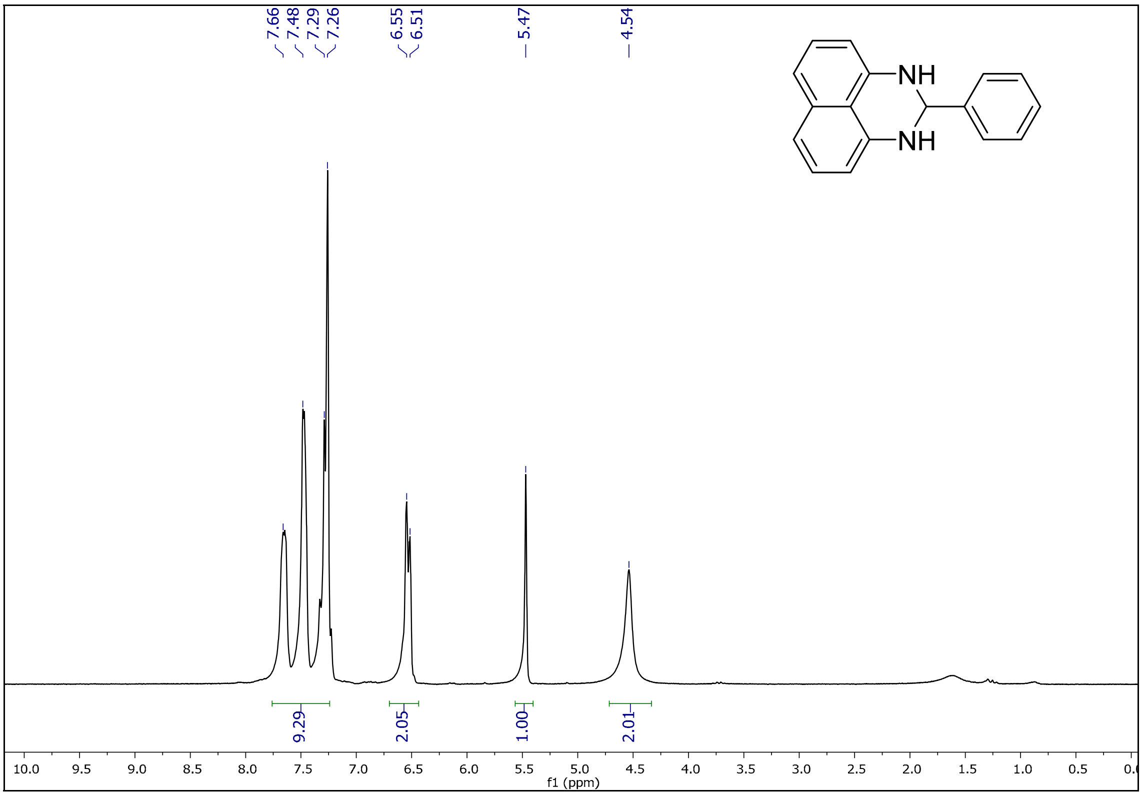

Figure S1. ^{1}H NMR spectrum (300 MHz, $CDCl_3$) of compound **3a**.

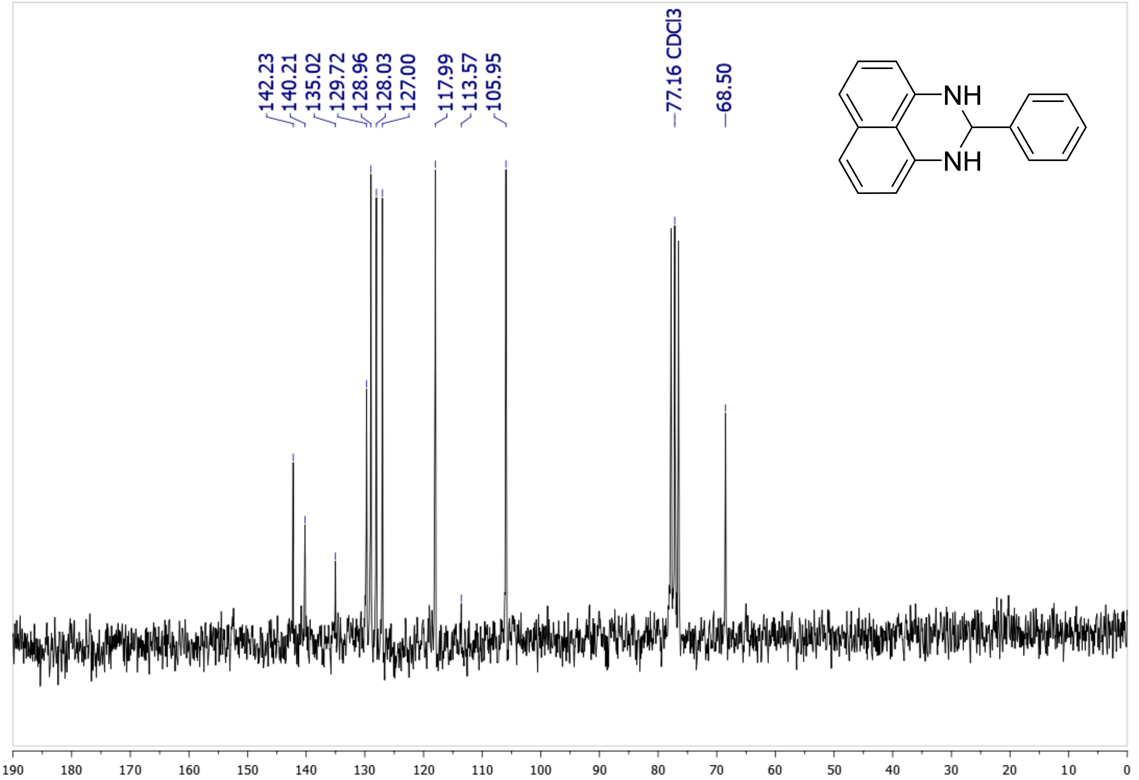

Figure S2. ^{13}C NMR spectrum (75 MHz, $CDCl_3$) of compound **3a**.

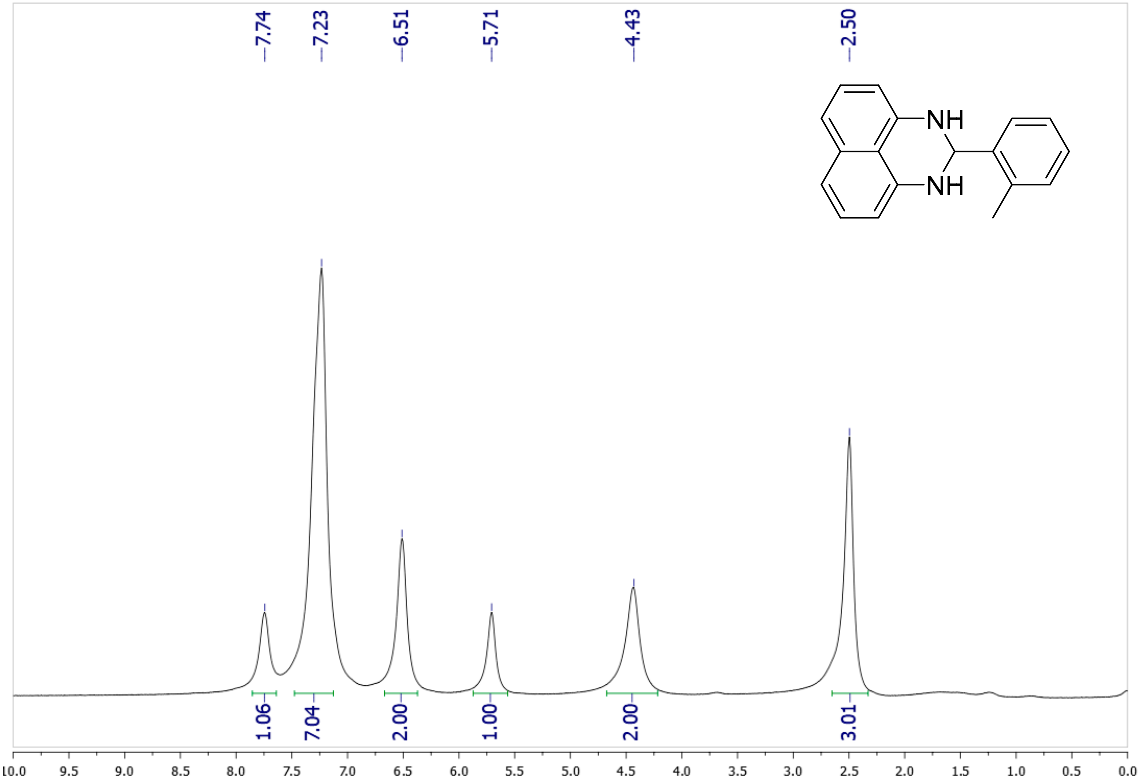

Figure S3. ^{1}H NMR spectrum (300 MHz, $CDCl_3$) of compound **3b**.

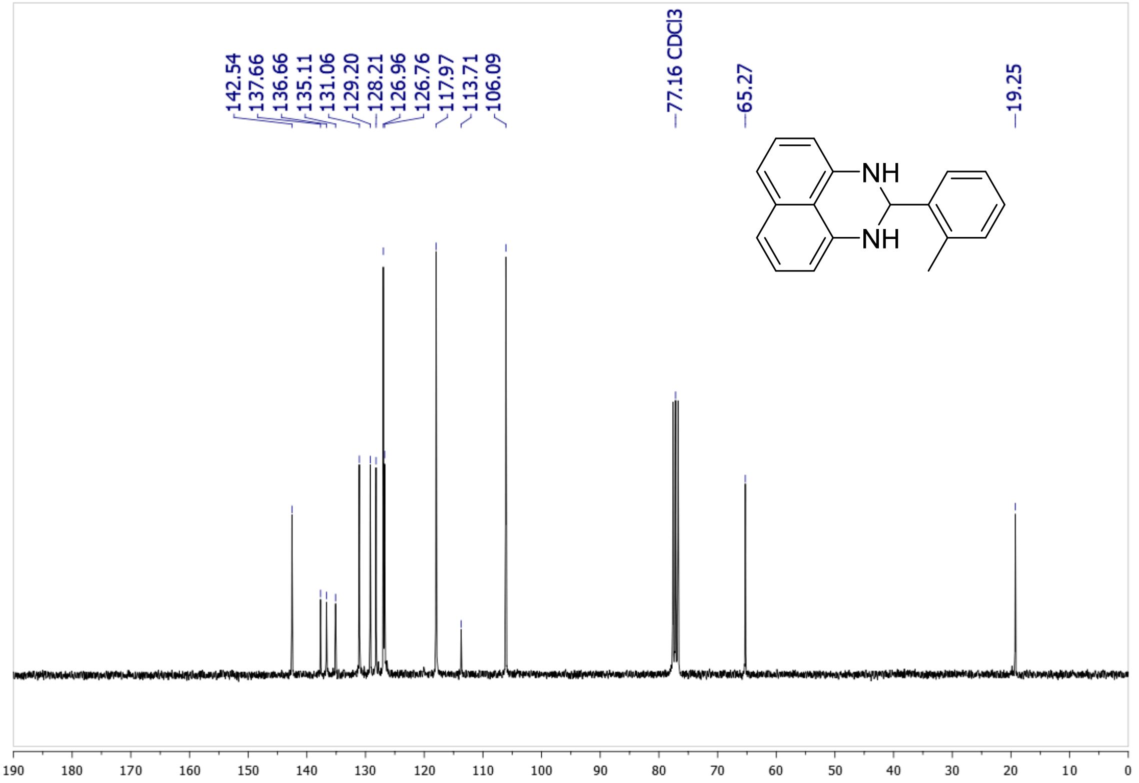

Figure S4. ^{13}C NMR spectrum (75 MHz, $CDCl_3$) of compound **3b**.

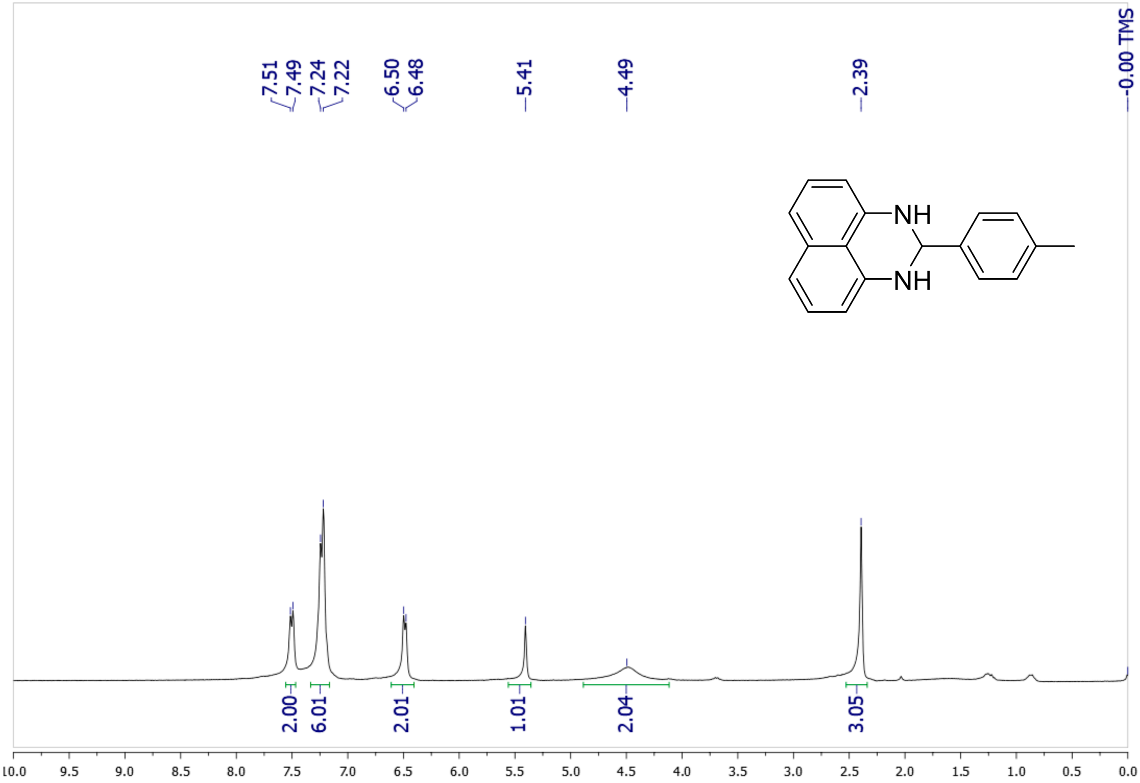

Figure S5. ^{1}H NMR spectrum (300 MHz, $CDCl_3$) of compound **3c**.

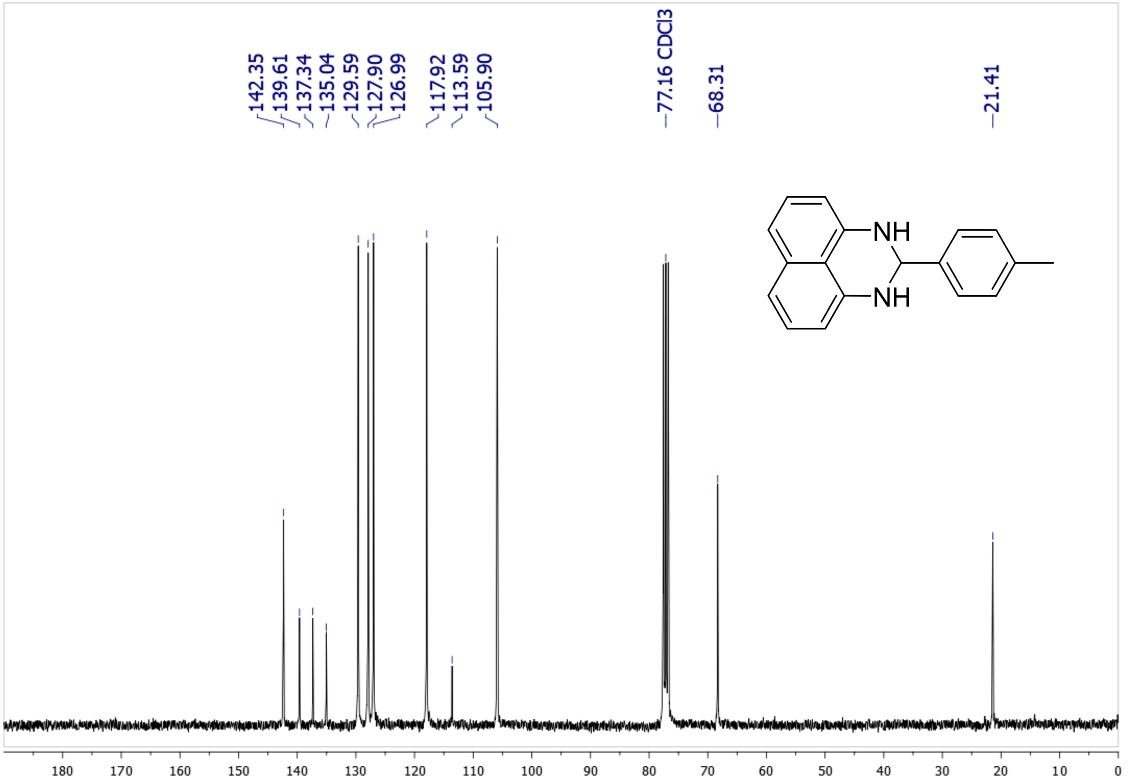

Figure S6. ^{13}C NMR spectrum (75 MHz, $CDCl_3$) of compound **3c**.

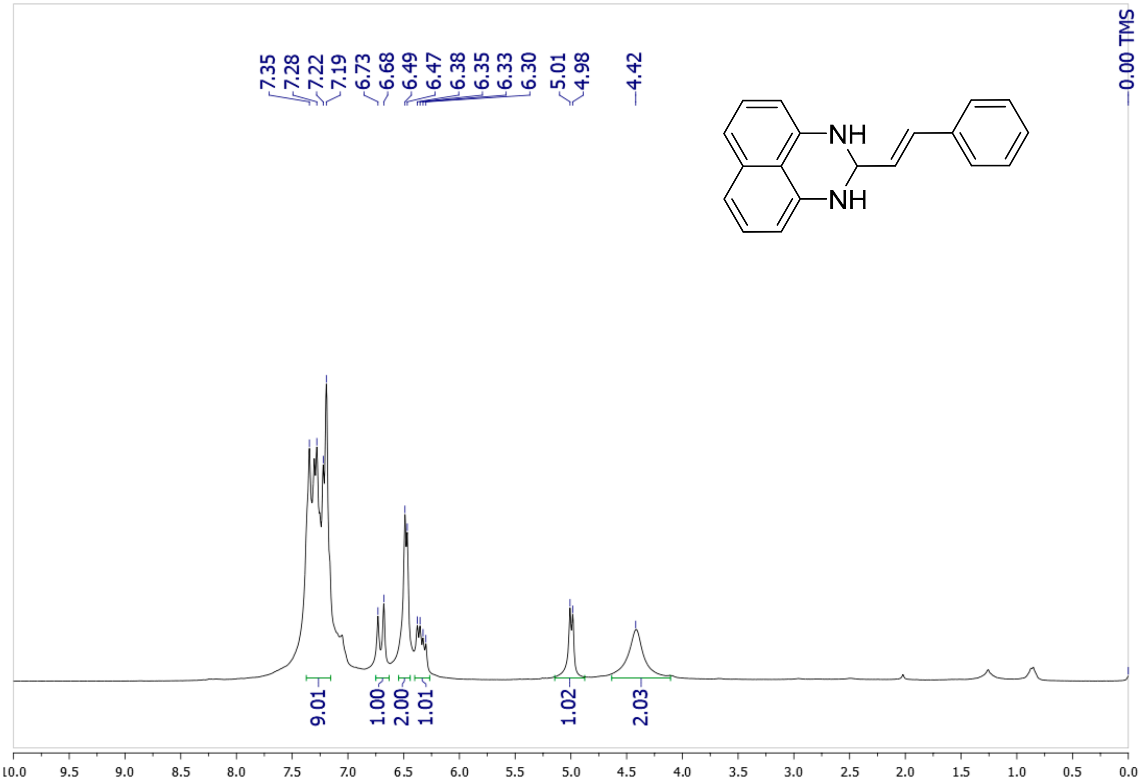

Figure S7. ^{1}H NMR spectrum (300 MHz, $CDCl_3$) of compound **3d**.

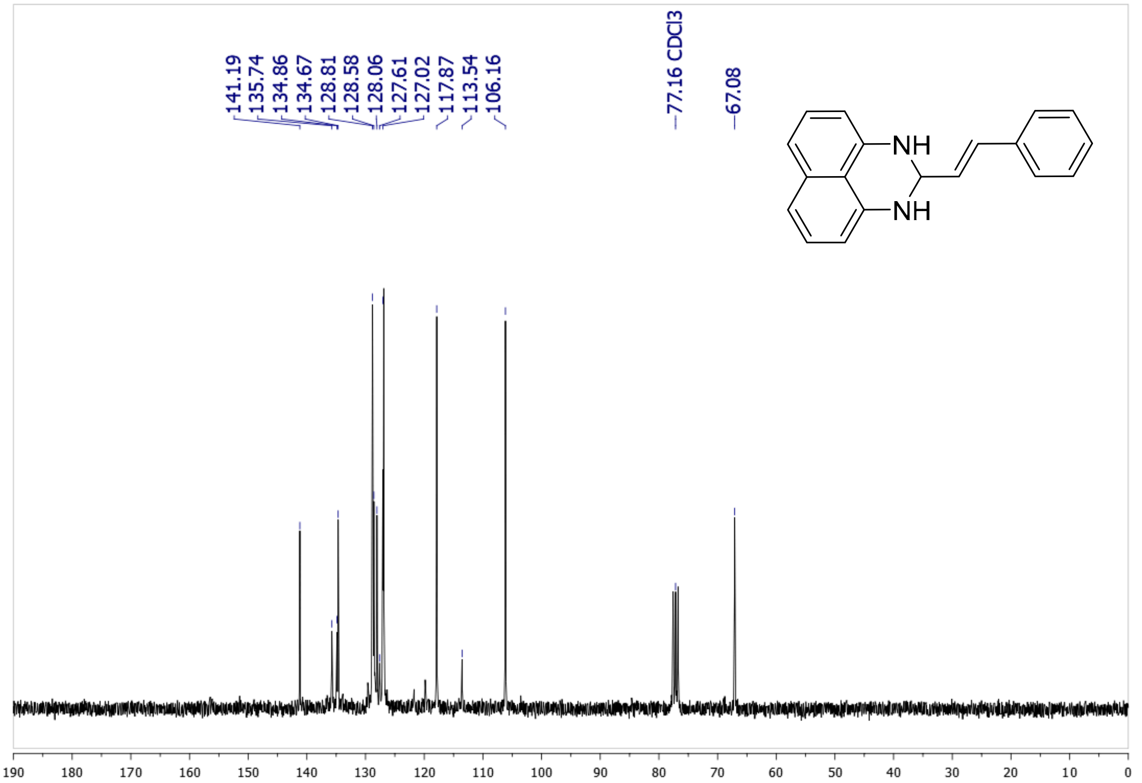

Figure S8. ^{13}C NMR spectrum (75 MHz, $CDCl_3$) of compound **3d**.

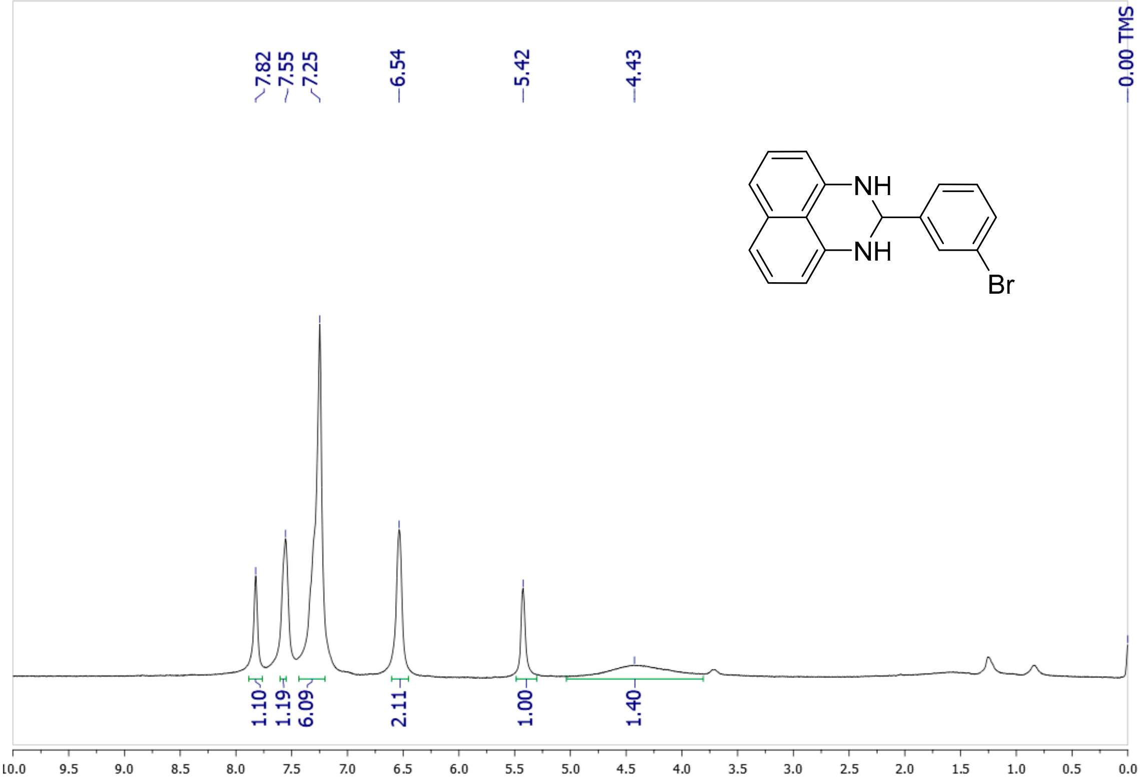

Figure S9. ^{1}H NMR spectrum (300 MHz, $CDCl_3$) of compound **3e**.

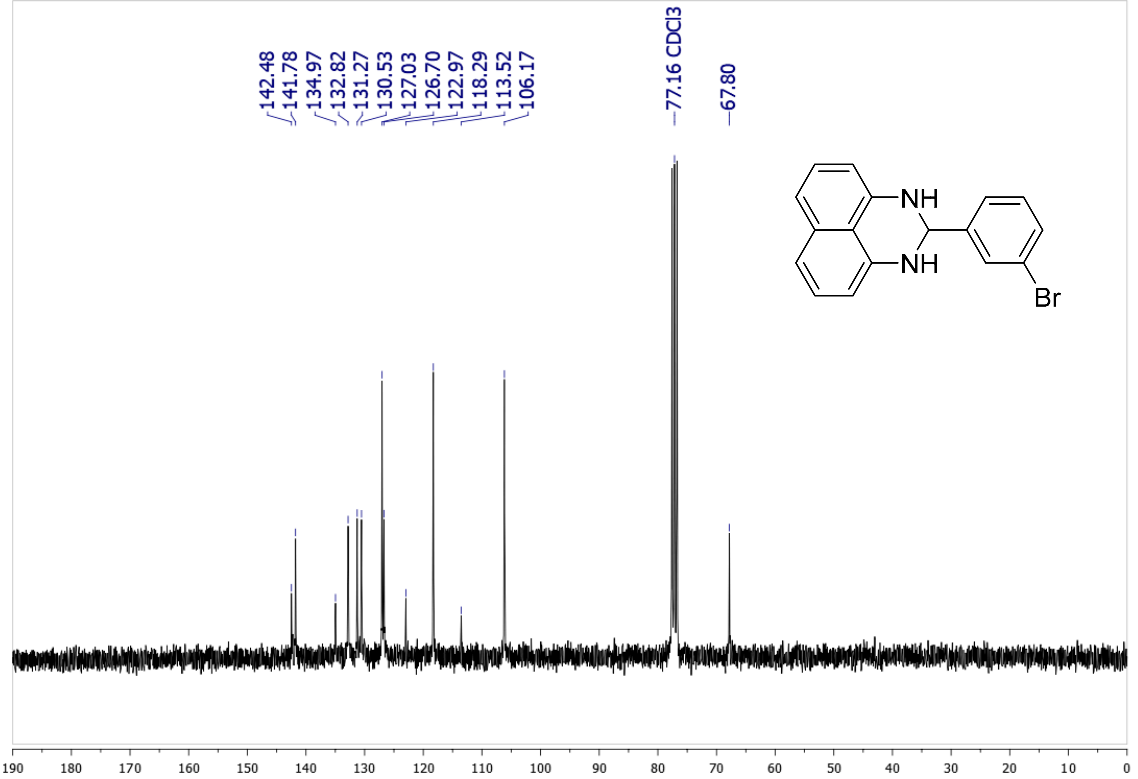

Figure S10. ^{13}C NMR spectrum (75 MHz, $CDCl_3$) of compound **3e**.

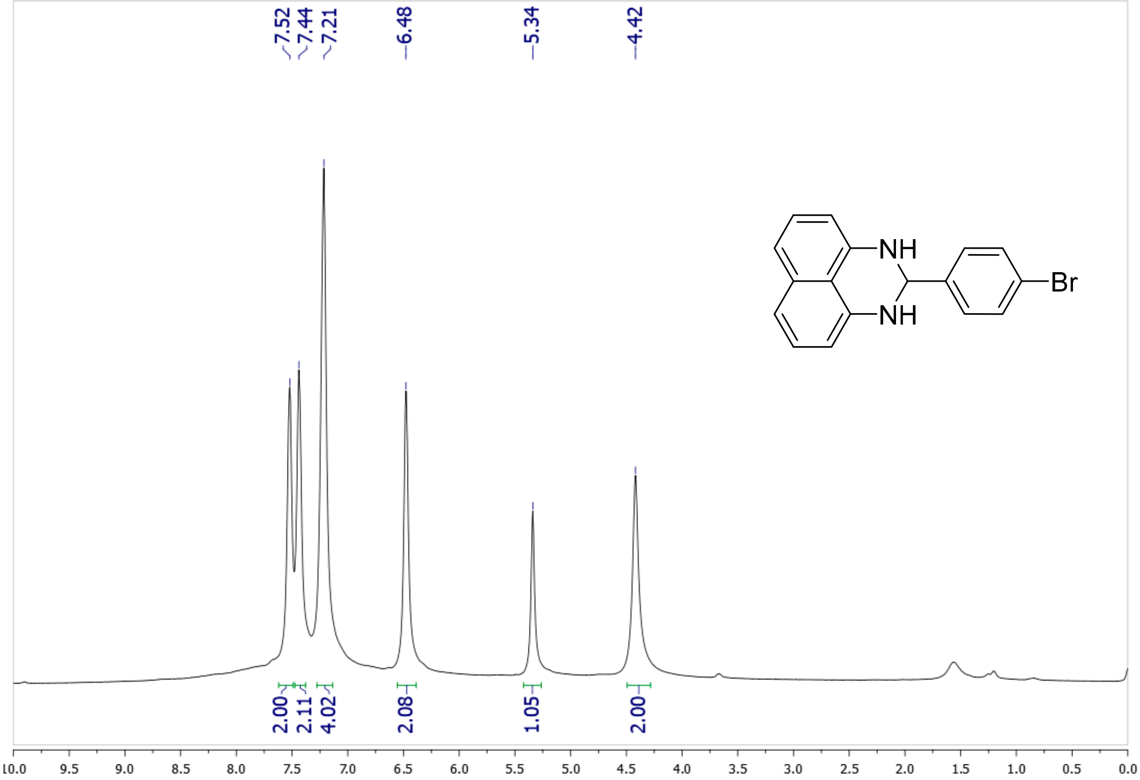

Figure S11. ^{1}H NMR spectrum (500 MHz, $CDCl_3$) of compound **3f**.

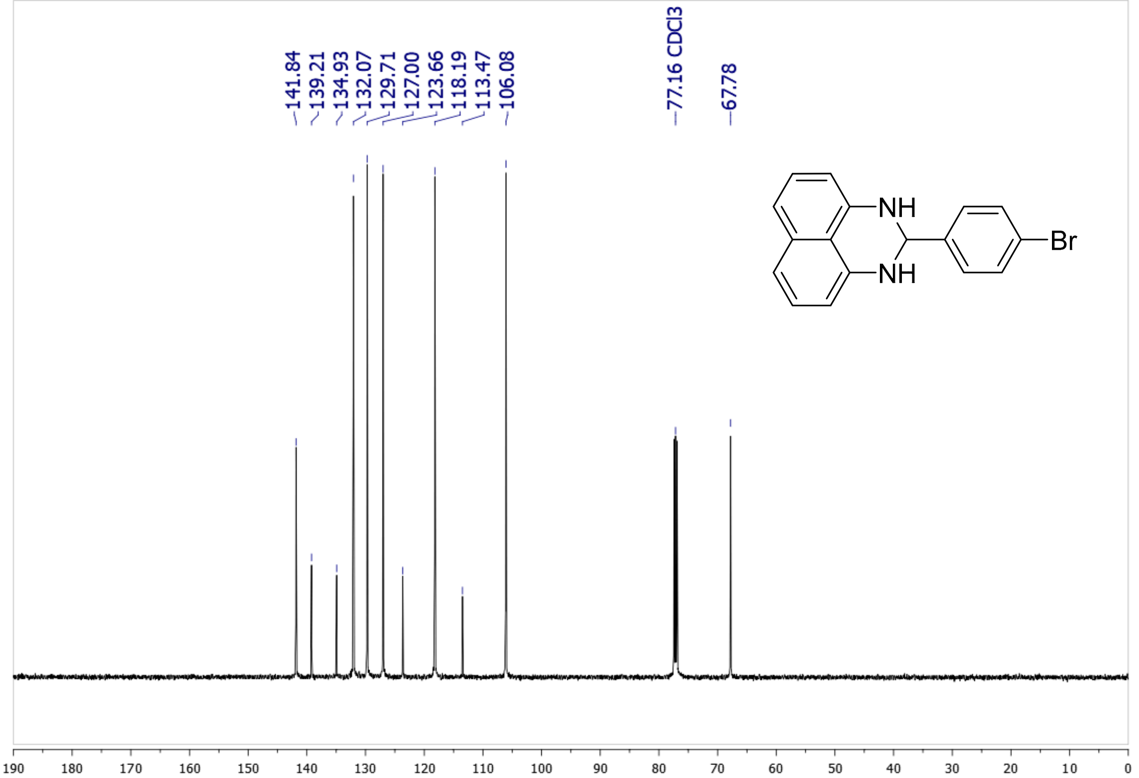

Figure S12. ^{13}C NMR spectrum (125 MHz, $CDCl_3$) of compound **3f**.

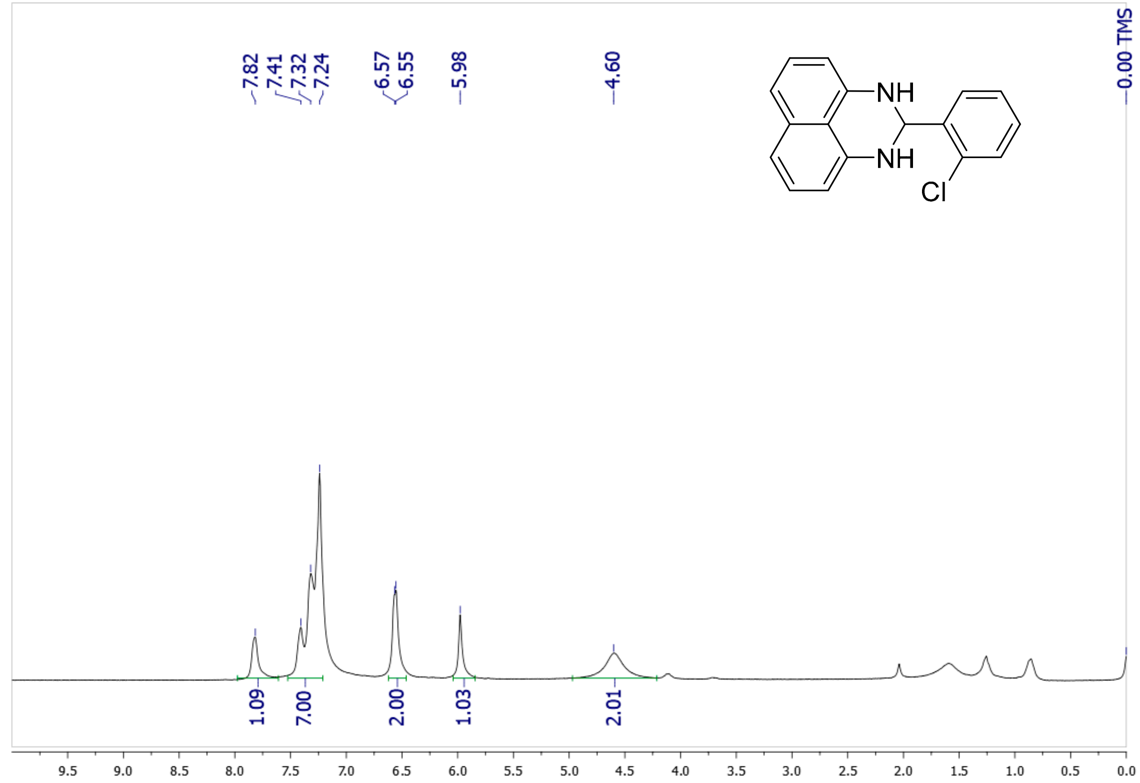

Figure S13. ^{1}H NMR spectrum (300 MHz, $CDCl_3$) of compound **3g**.

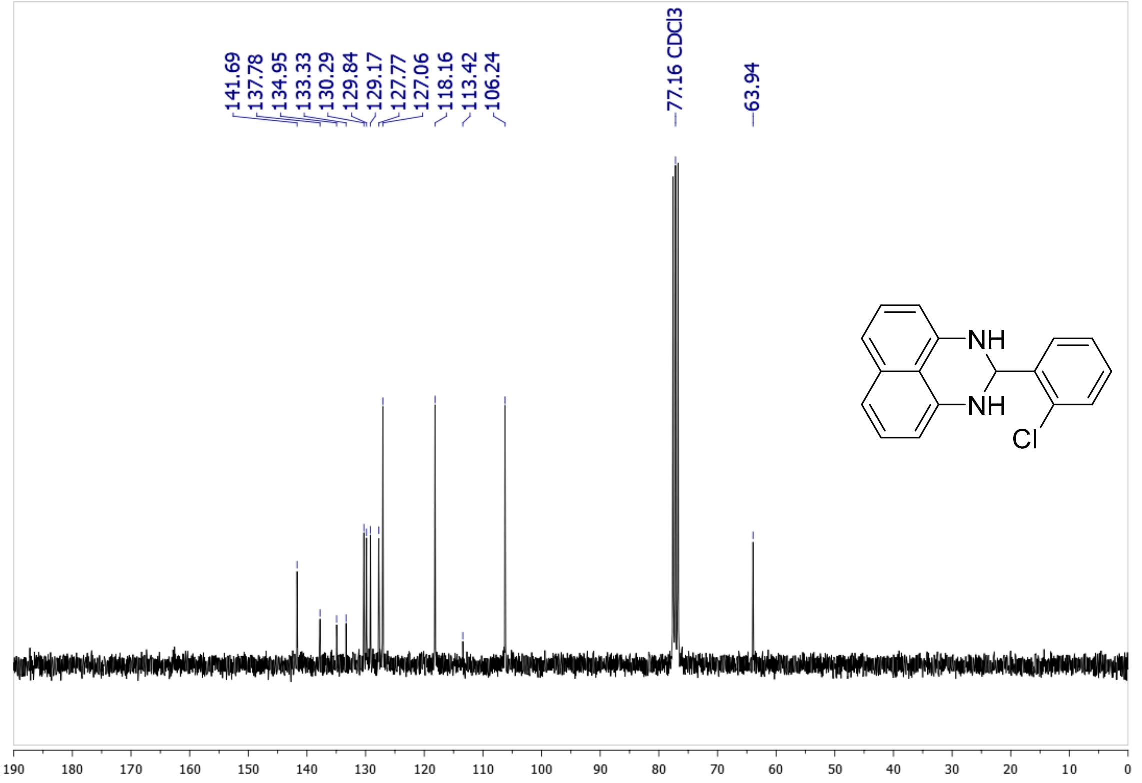

Figure S14. ^{13}C NMR spectrum (75 MHz, $CDCl_3$) of compound **3g**.

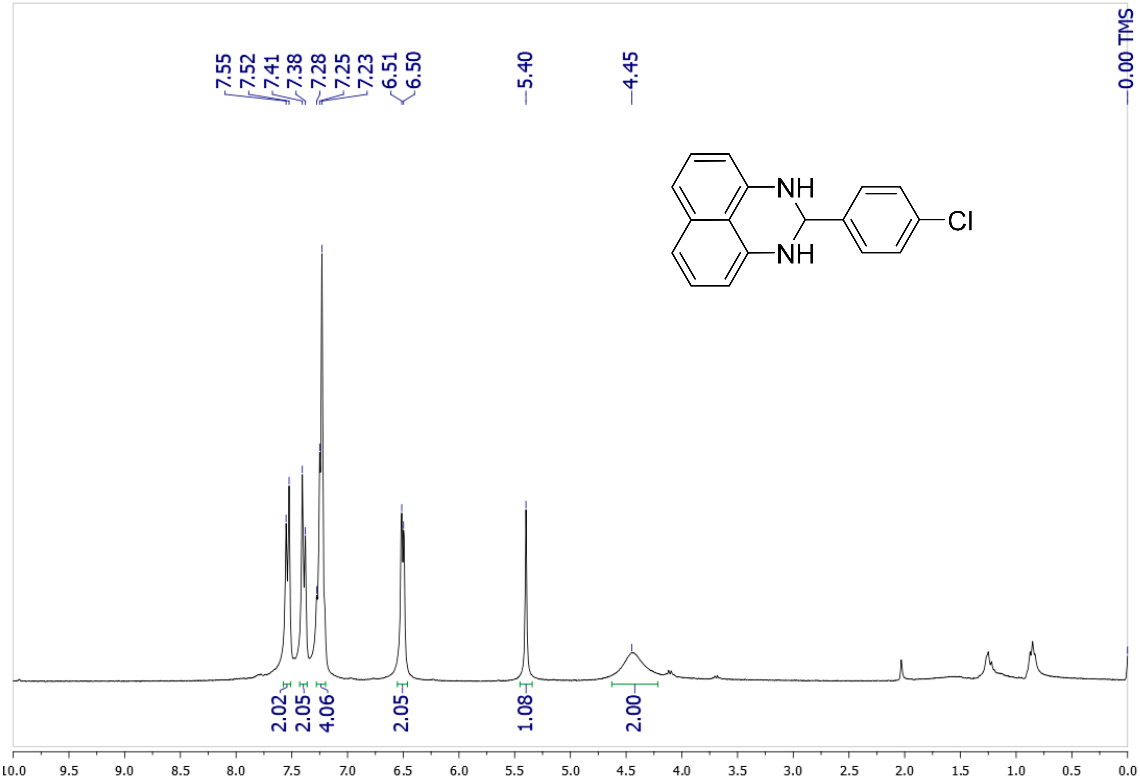

Figure S15. ^{1}H NMR spectrum (300 MHz, $CDCl_3$) of compound **3h**.

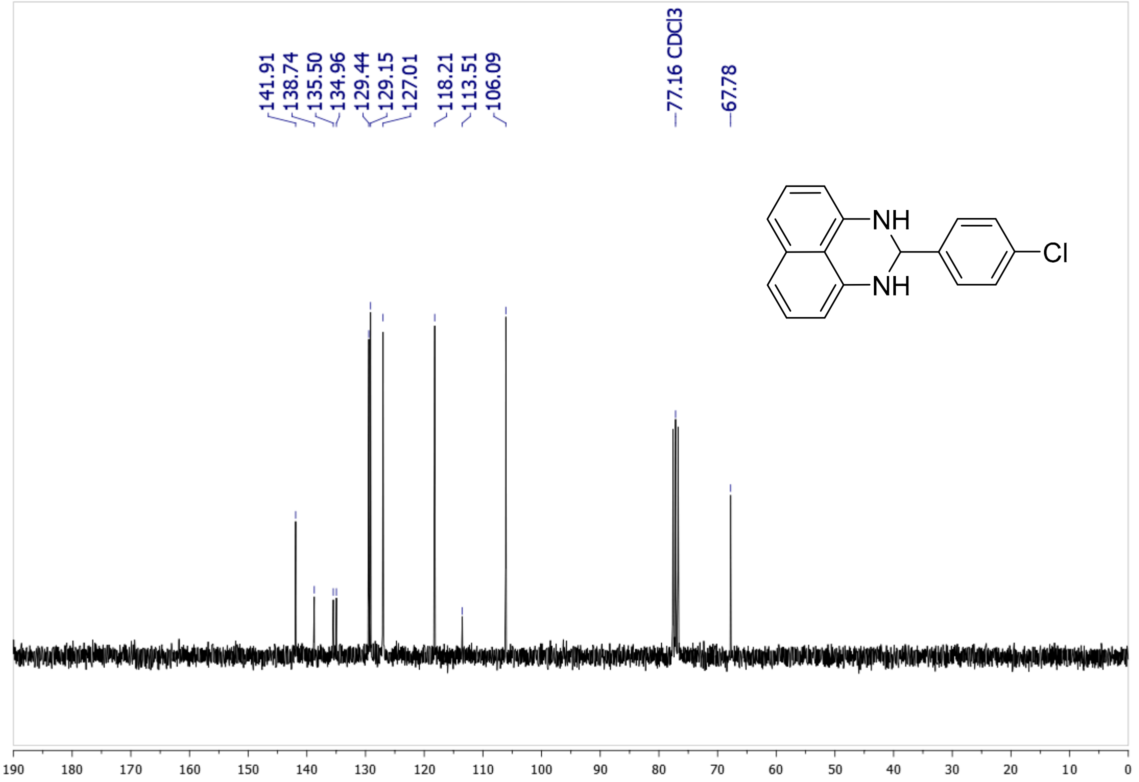

Figure S16. ^{13}C NMR spectrum (75 MHz, $CDCl_3$) of compound **3h**.

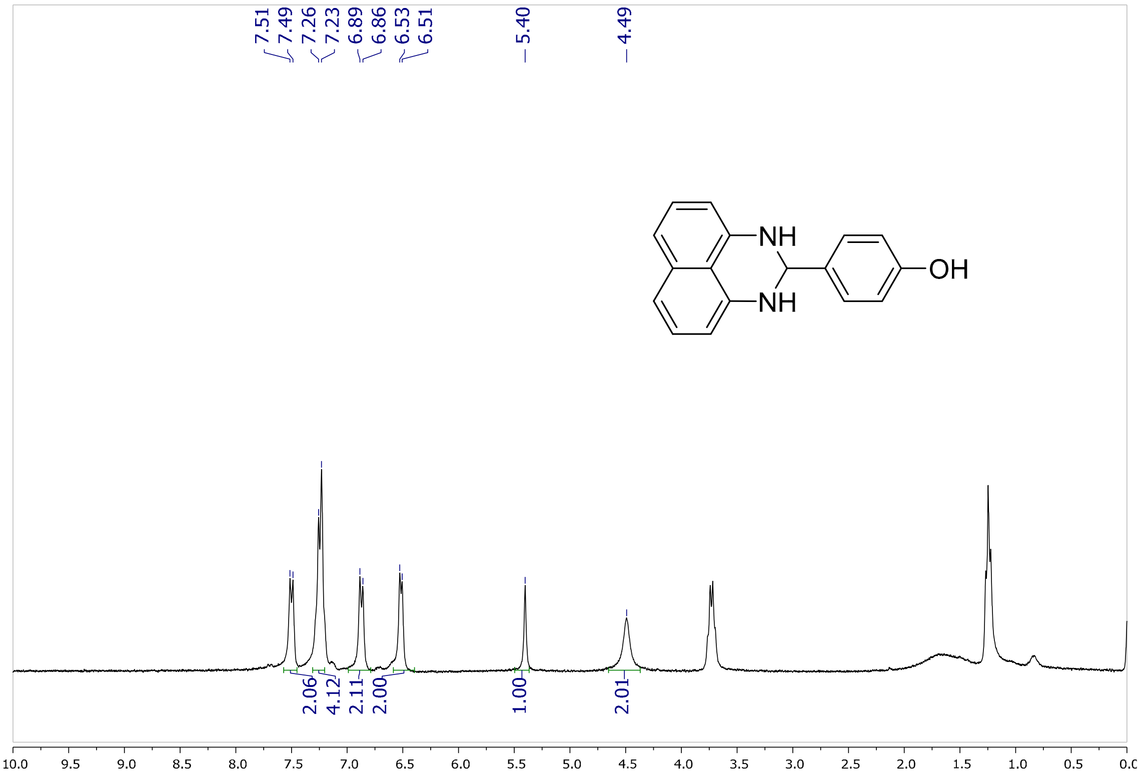

Figure S17. ^{1}H NMR spectrum (300 MHz, $CDCl_3$) of compound **3i**.

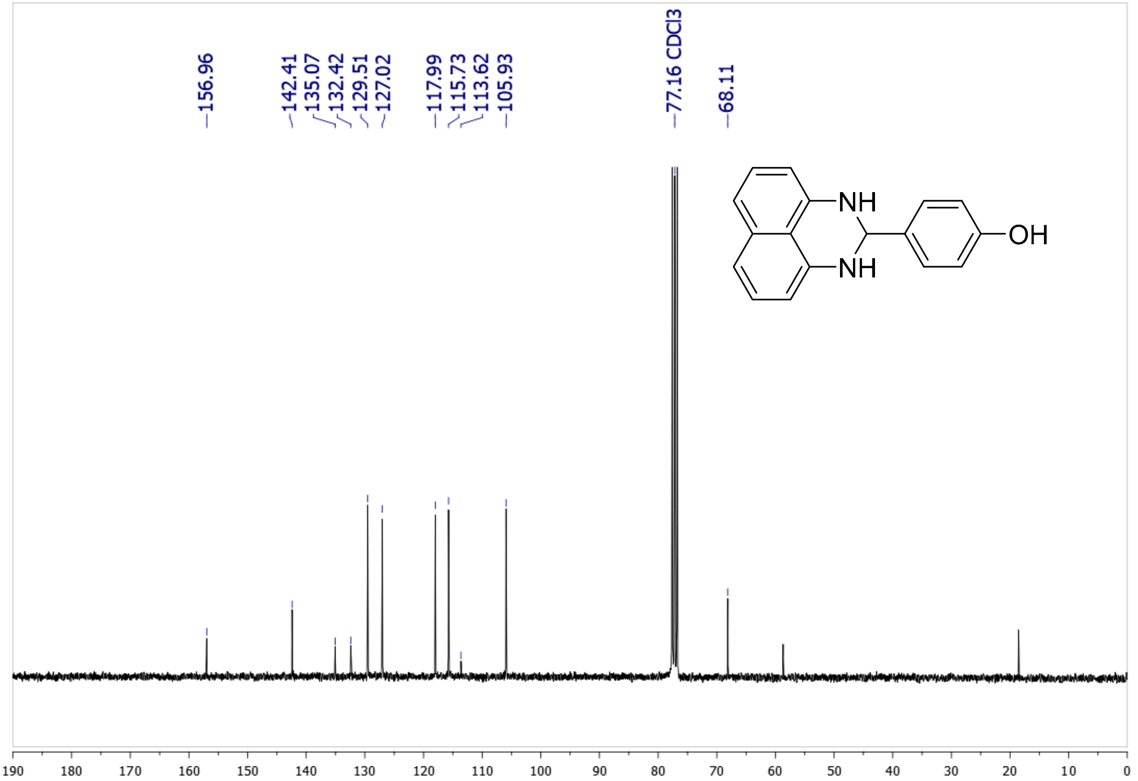

Figure S18. ^{13}C NMR spectrum (75 MHz, $CDCl_3$) of compound **3i**.

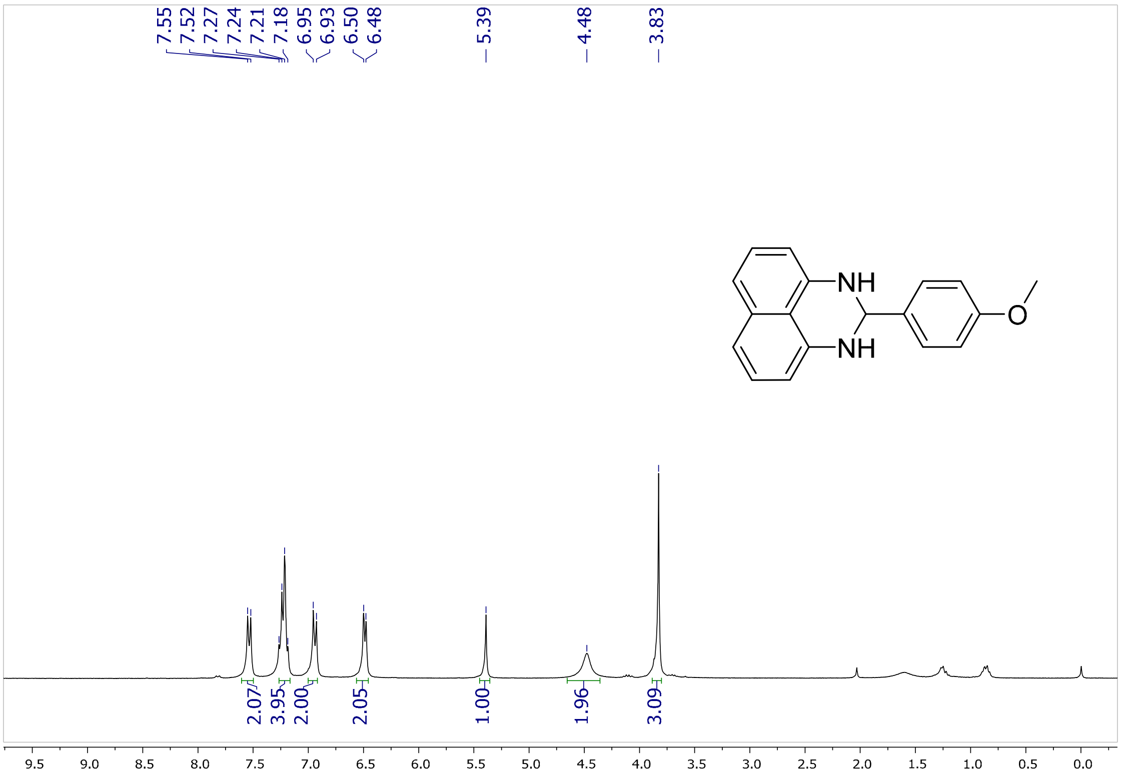

Figure S19. ^{1}H NMR spectrum (300 MHz, $CDCl_3$) of compound **3j**.

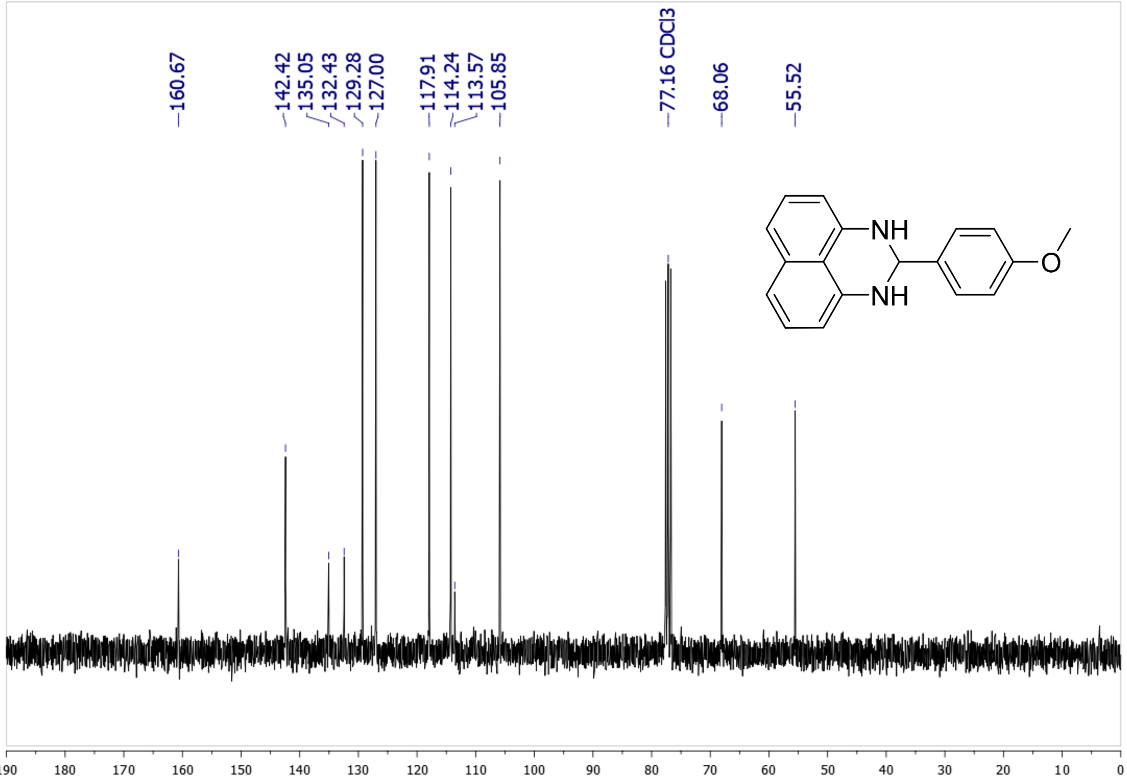

Figure S20. ^{13}C NMR spectrum (75 MHz, $CDCl_3$) of compound **3j**.

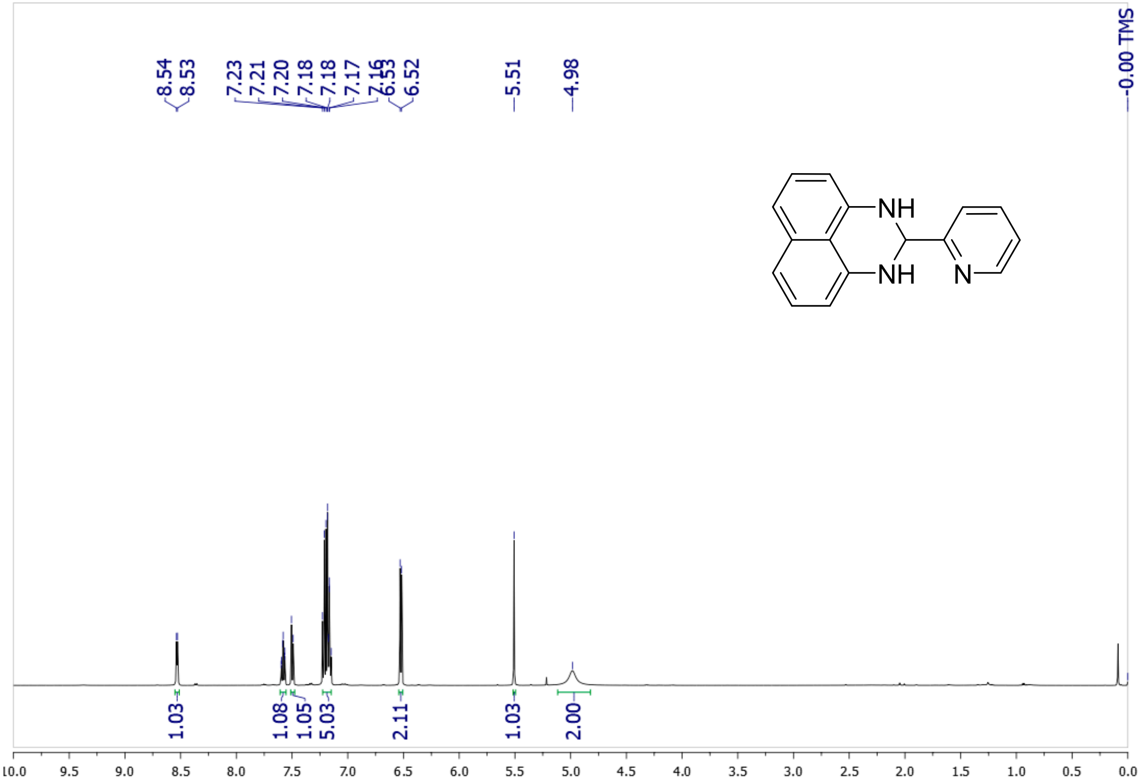

Figure S21. ^{1}H NMR spectrum (500 MHz, $CDCl_3$) of compound **3k**.

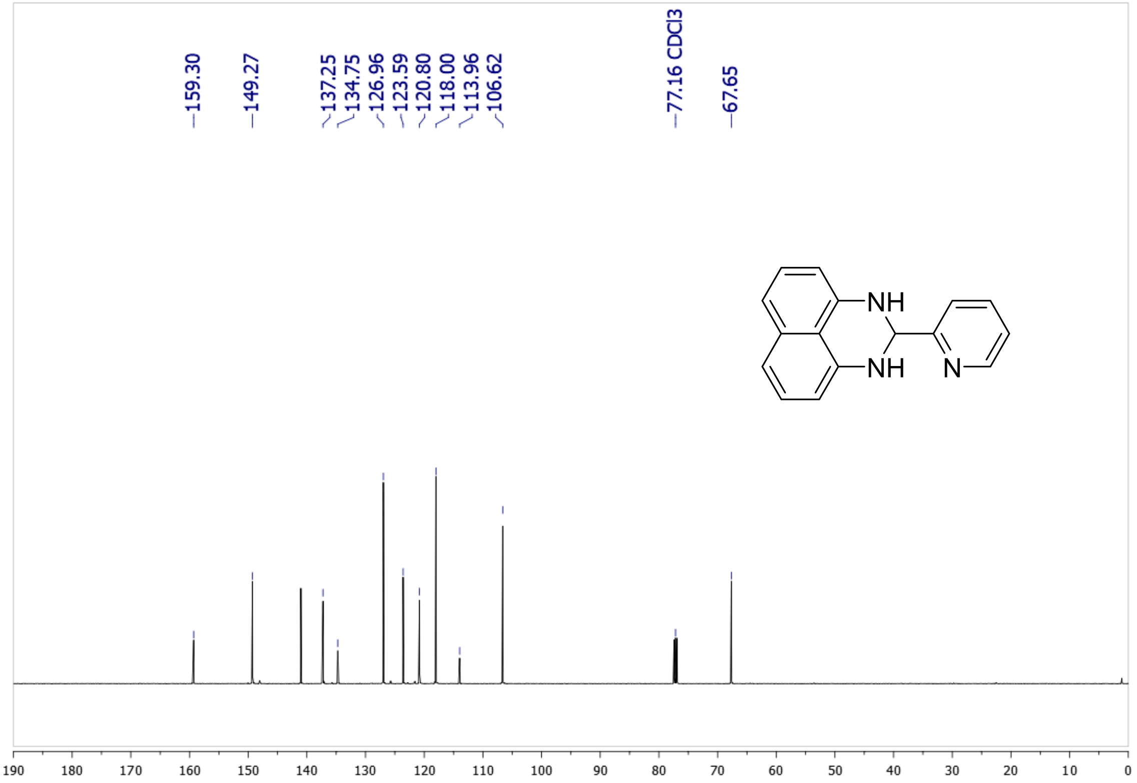

Figure S22. ^{13}C NMR spectrum (126 MHz, $CDCl_3$) of compound **3k**.

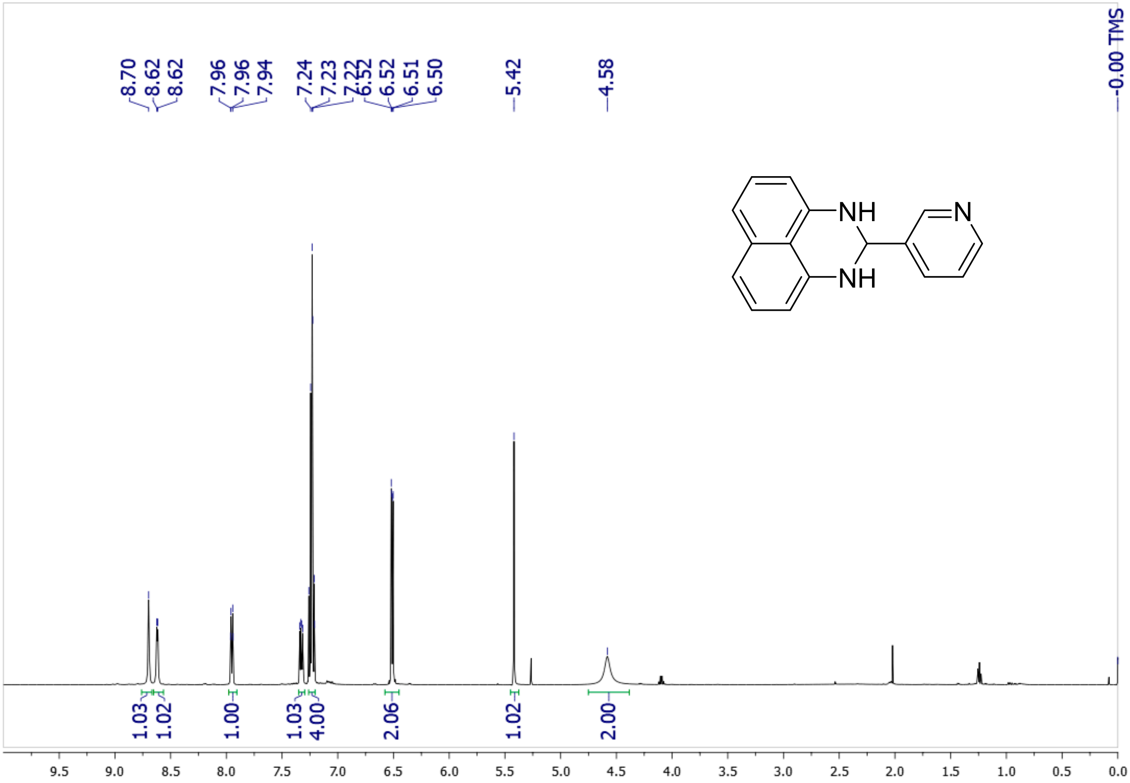

Figure S23. ^{1}H NMR spectrum (500 MHz, $CDCl_3$) of compound **3l**.

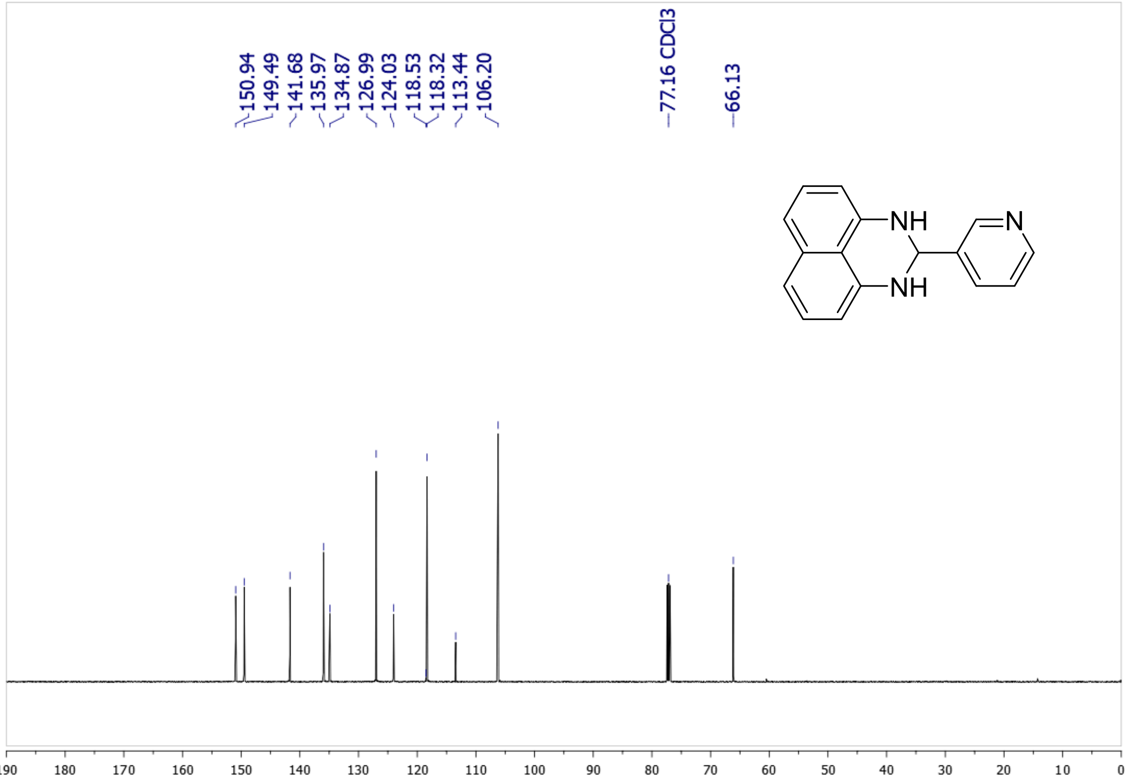

Figure S24. ^{13}C NMR spectrum (126 MHz, $CDCl_3$) of compound **3l**.

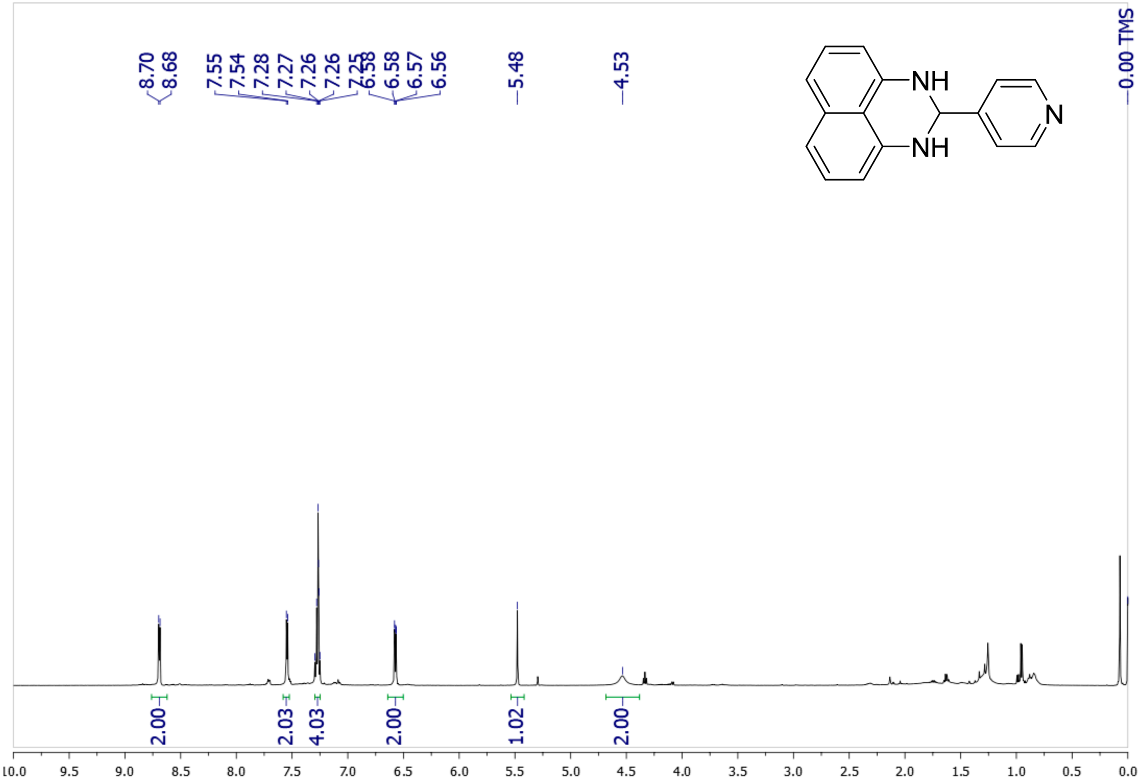

Figure S25. ^{1}H NMR spectrum (500 MHz, $CDCl_3$) of compound **3m**.

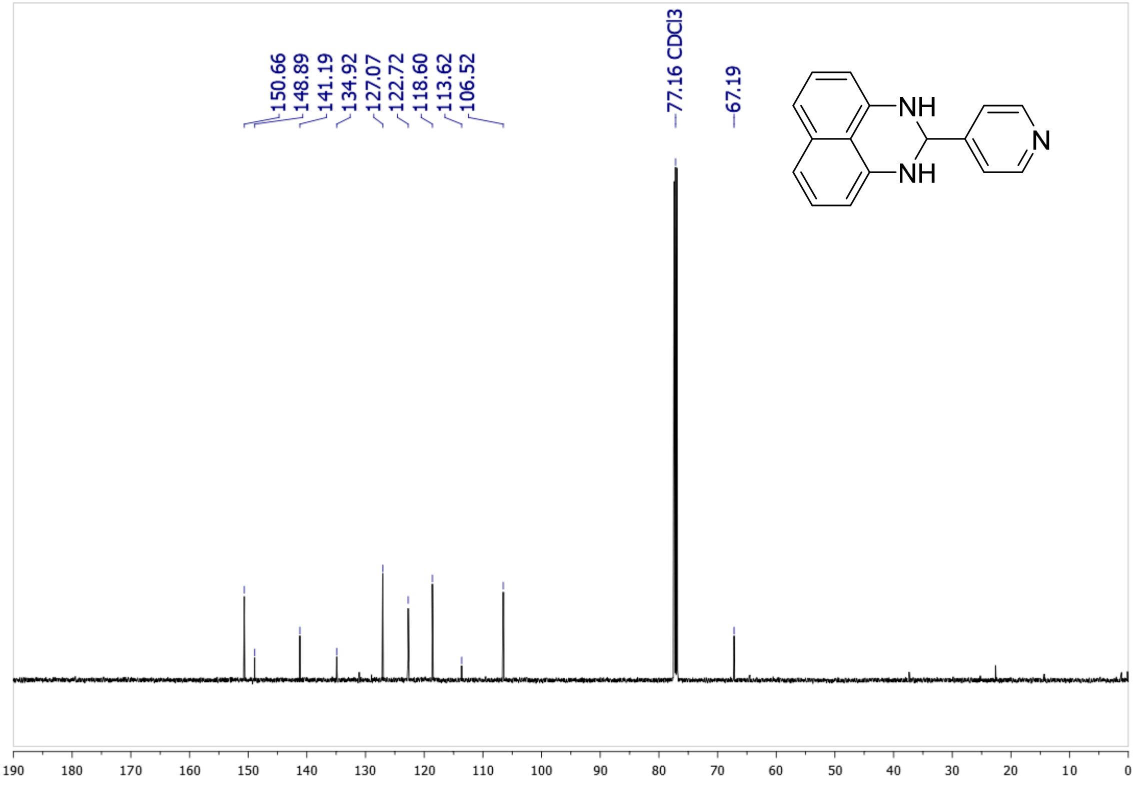

Figure S26. ^{13}C NMR spectrum (126 MHz, $CDCl_3$) of compound **3m**.

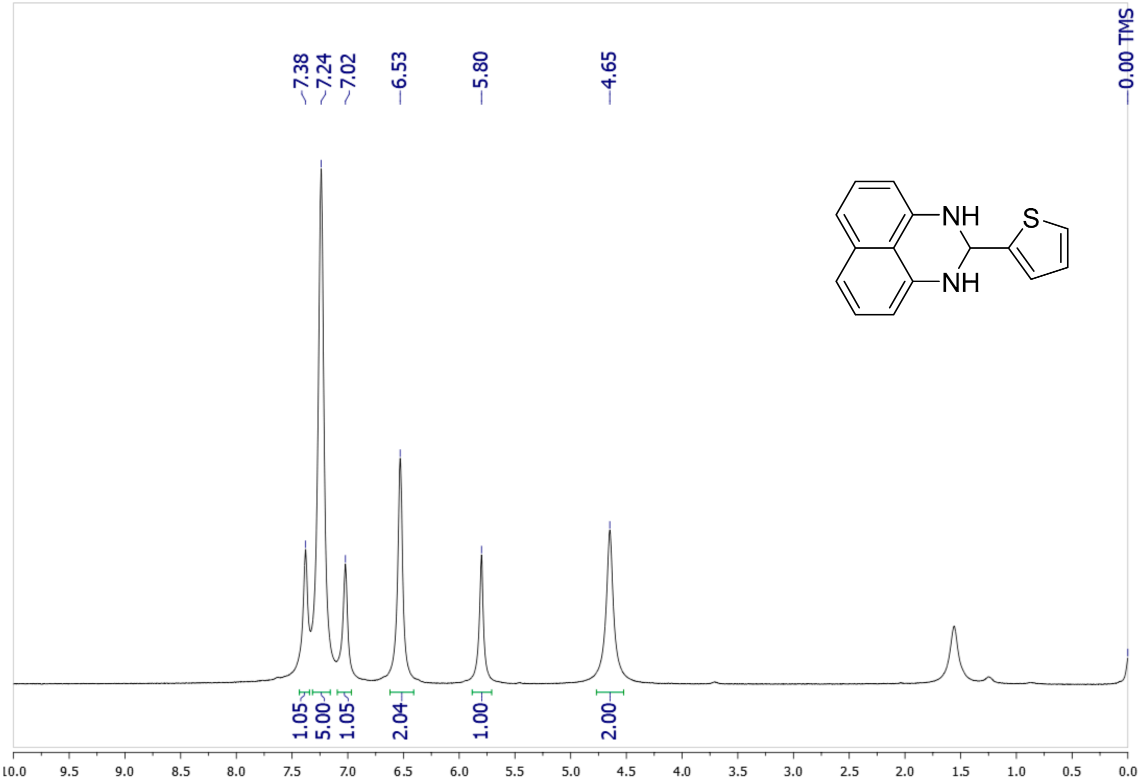

Figure S27. ^{1}H NMR spectrum (500 MHz, $CDCl_3$) of compound **3n**.

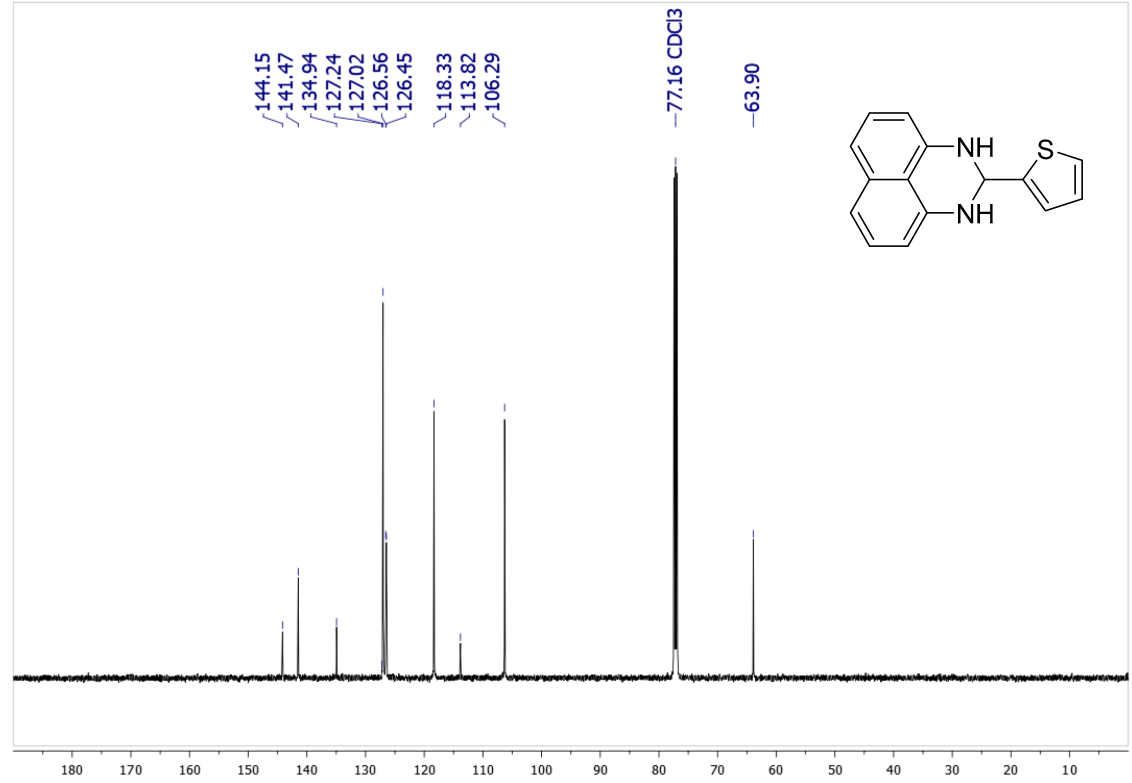

Figure S28. ^{13}C NMR spectrum (126 MHz, $CDCl_3$) of compound **3n**.

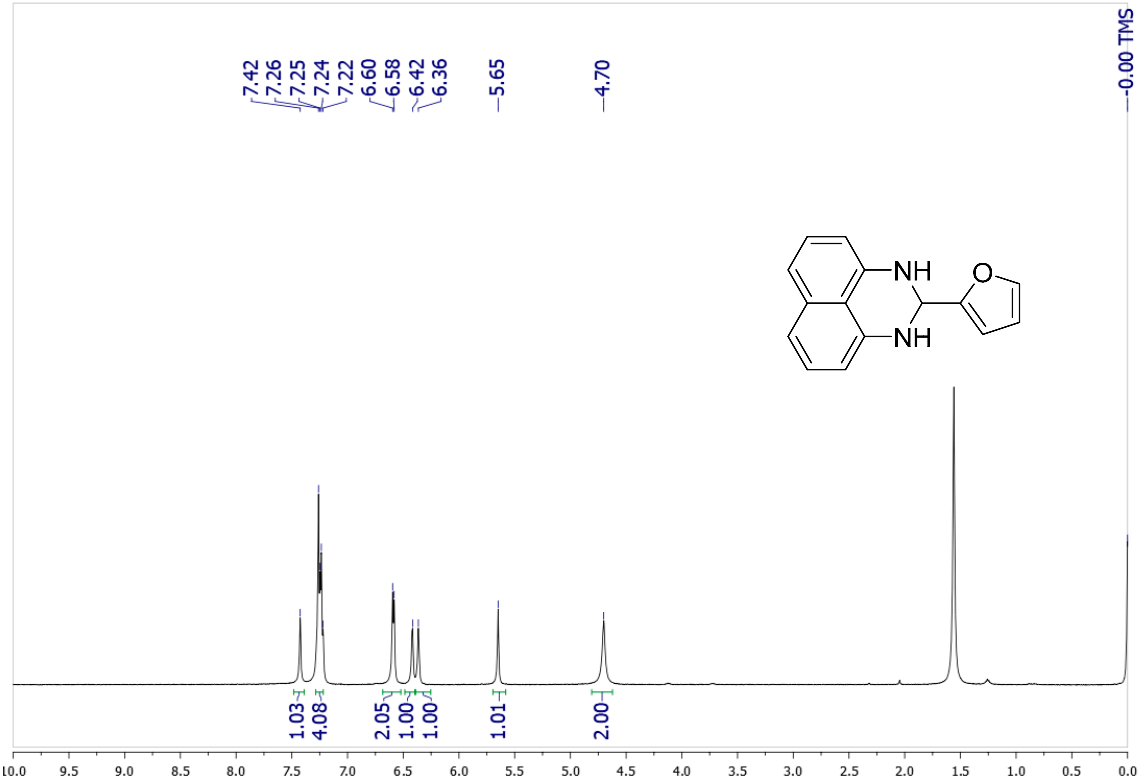

Figure S29. ^{1}H NMR spectrum (500 MHz, $CDCl_3$) of compound **3o**.

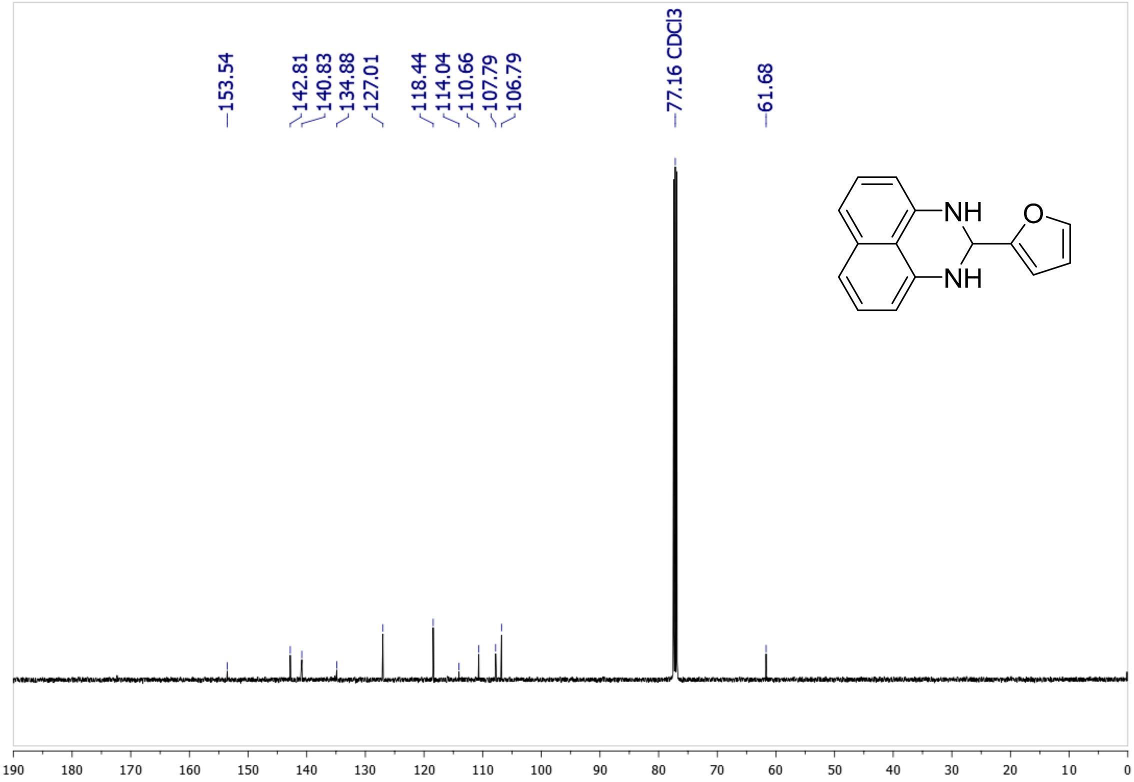

Figure S30. ^{13}C NMR spectrum (126 MHz, $CDCl_3$) of compound **3o**

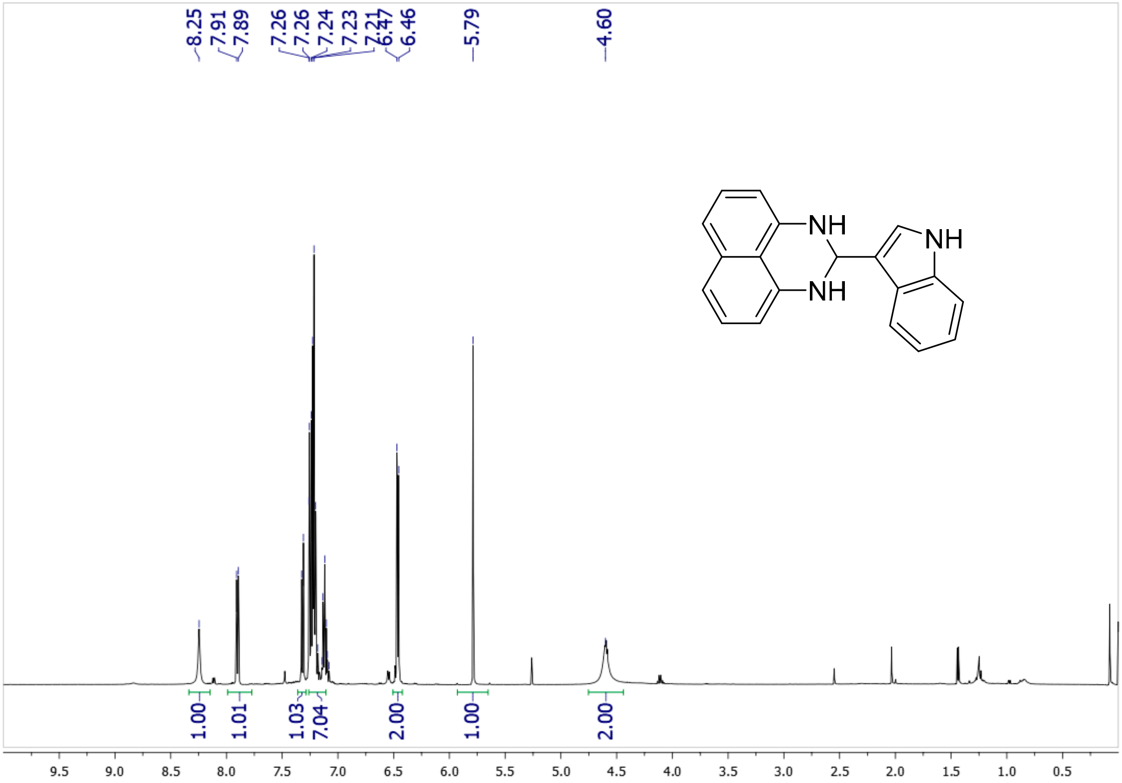

Figure S31. ^{1}H NMR spectrum (500 MHz, $CDCl_3$) of compound **3p**.

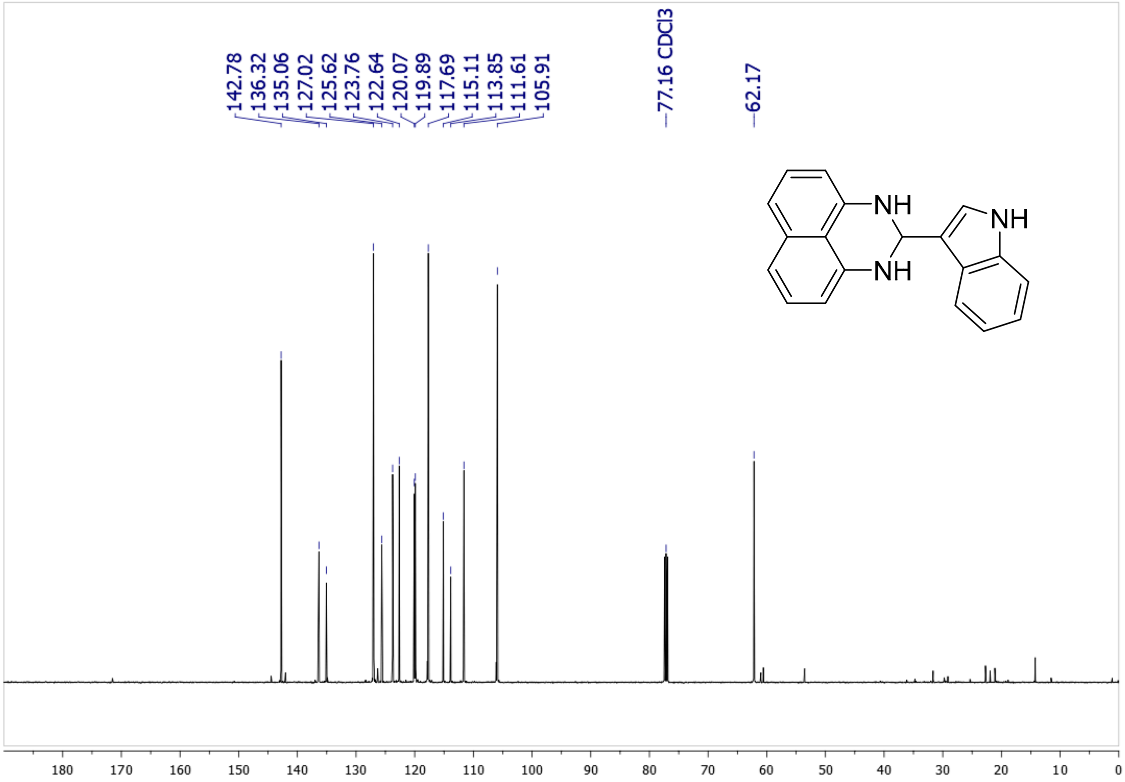

Figure S32. ^{13}C NMR spectrum (126 MHz, $CDCl_3$) of compound **3p**.
